# Pan-African hybridization of PfSPZ increases antigenic diversity and replicative capacity for malaria vaccine design

**DOI:** 10.1172/jci.insight.207272

**Published:** 2026-08-18

**Authors:** Lucia Pazzagli, Bethany Jenkins, Ankit Dwivedi, Asha Patil, Yonas Abebe, Tales V. Pascini, Urvashi Rai, Priya Gupta, Nastaran Rezakhani, Chakshu Gandhi, Yiwei Yang, Sudhir Kumar, Mohd Kamil, Gigliola Zanghí, Manuel Llinás, Stephen L. Hoffman, Joana C. Silva, Ashley M. Vaughan, B. Kim Lee Sim

**Affiliations:** 1 Center for Global Infectious Disease Research, Seattle Children’s Research Institute, Seattle, Washington, USA.; 2 Sanaria Inc., Rockville, Maryland, USA.; 3 Institute for Genome Sciences, University of Maryland, Baltimore, Maryland, USA.; 4 Department of Biochemistry and Molecular Biology, Department of Chemistry, Huck Center for Malaria Research, The Pennsylvania State University, University Park, Pennsylvania, USA.; 5 Global Health and Tropical Medicine, Instituto de Higiene e Medicina Tropical, Universidade NOVA de Lisboa, Lisbon, Portugal.; 6 Department of Pediatrics and; 7 Department of Global Health, University of Washington, Seattle, Washington, USA.

**Keywords:** Immunology, Infectious disease, Malaria, Parasitology, Vaccines

## Abstract

*Plasmodium falciparum* sporozoite (PfSPZ) vaccines, composed of aseptic, purified, live parasites that arrest during or just after liver-stage development, show excellent safety and efficacy in humans. They can induce complete protection against Pf infection, mediated primarily by cellular immune responses against parasite antigens expressed in hepatocytes. Current PfSPZ vaccines rely on the West African PfNF54 parasite, which uniquely produces high numbers of PfSPZ in mosquitoes, facilitating manufacturing efficiency. However, PfNF54 has relatively low hepatocyte infectivity, limiting potency. We created hybrid pan-African Pf strains by genetically crossing PfNF54 with East African Pf strains. The hybrid, AV27, was selected for development based on balanced contribution of parental genomes, high PfSPZ production, and high liver-stage infectivity. As compared with NF54-based PfSPZ vaccines, we expect AV27-based vaccines will have greater and broader efficacy at lower doses due to higher liver-stage infectivity and inclusion of unique East African CD8^+^ T cell epitopes.

## Abstract 2

A new malaria vaccine strain combines parasite diversity from across Africa with greater infectivity, offering the potential for broader, more effective protection at lower doses.

## Introduction

There were more clinical cases and deaths caused by malaria in 2024 than in 2015 despite more than $4.5 billion in annual investment in malaria control efforts (1). A highly effective malaria vaccine is needed (2). *Plasmodium falciparum* (Pf) sporozoite (SPZ) vaccines are composed of live, aseptic, purified PfSPZ that arrest during or just after liver-stage development. PfSPZ vaccines are powerful immunogens, providing sterilizing protection against field-transmitted Pf and controlled human malaria infection (CHMI) (3).

PfSPZ are the only injectable malaria vaccines that have provided 100% vaccine efficacy (VE) against homologous or heterologous CHMI (4, 5). In addition, PfSPZ vaccines have induced greater than 50% VE for up to 21 months in West African adults without boosting, 57% VE against pregnancy malaria in West African women for up to 21 months (6), high-level protection during the first year of life in infants born to mothers immunized before conception (7), and 56% VE for 6 months in malaria-naive Indonesian soldiers against highly divergent Pf in Papua, Indonesia (8). Additionally, immunization by mosquito bite with late liver stage–arresting replication-competent (LARC) PfSPZ (9–11) is more protective than is immunization with early-arresting replication-deficient (EARD) PfSPZ (10). This is likely due to the increased antigenic load of the LARC parasite, which expresses about 3,000 more proteins than EARD parasites and replicates up to 50,000 times, while EARD parasites do not replicate.

All current PfSPZ vaccines are based on the West African isolate, NF54 (isolated in 1979) (12, 13), selected for its robust PfSPZ production in aseptic *Anopheles stephensi* mosquitoes (>100,000 PfSPZ per mosquito). The efficacy of PfSPZ vaccines is thought to be primarily mediated by protective CD8^+^ T cell epitopes expressed by Pf liver-stage parasites (14). Target T cell epitopes likely vary among Pf isolates, and this variation increases with parasite genetic distance from West African NF54 (15, 16) and could reduce VE provided by PfNF54 vaccines in other parts of Africa (17). Moreover, NF54 exhibits up to an 8-fold lower liver-stage infectivity for primary human hepatocytes in vitro compared with more recently isolated Pf strains (18, 19), which likely leads to suboptimal VE of NF54-based PfSPZ vaccines.

Enhancement of VE against divergent Pf strains could potentially be achieved by separate manufacturing and coadministration of PfSPZ derived from parasite isolates originating from distinct geographic regions. Mixing of strains has been used in viral, bacterial, and parasite human and veterinary vaccines (20–24). PfSPZ from the Brazilian clone Pf7G8 have been manufactured as Sanaria PfSPZ Challenge (7G8) for CHMI at 3.2 × 10^3^ PfSPZ per dose (5, 25, 26). However, mosquitoes produce PfSPZ (7G8) at 5–40 times lower rates than NF54, making PfSPZ (7G8) cost-prohibitive for vaccine manufacturing. In addition, all other Pf strains assessed have had similarly low PfSPZ during current good manufacturing practice–compliant (cGMP-compliant) production in mosquitoes.

To overcome the limitations of NF54 PfSPZ and the poor PfSPZ production of non-NF54 Pf strains, we hypothesized that genetically crossing East African Pf field isolates with NF54 would yield pan-African hybrid progeny with balanced expression of multiple protective epitopes from both parental strains, preserved high PfSPZ production of West African NF54, and the high liver-stage infectivity of the recently isolated East African partner. We report that this has been accomplished, providing the foundation for a next-generation PfSPZ vaccine with inferred improved VE at reduced cost as compared with the current genetically attenuated PfSPZ-LARC2 vaccine (NF54) carrying deletions in *PfMei2* and *PfLINUP* (27).

## Results

### Recent field isolates produce fewer PfSPZ in A. stephensi mosquitoes than NF54.

To support sustainable PfSPZ manufacturing, parasite strains need to be adapted to in vitro culture to produce mature sexual stage V gametocytes, which are fed to female *Anopheles* *stephensi* mosquitoes to initiate the mosquito stage of the parasite life cycle (28–30). However, in vitro gametocyte production is often associated with low yields, leading to poorly infected mosquitoes (31). An outlier to this observation is the well-established West African NF54 parasite strain, which easily generates infectious gametocytes in vitro yielding high numbers of PfSPZ in *A.* *stephensi* salivary glands. Based on this unique attribute, NF54 has been the only Pf strain utilized in the efficient manufacturing of PfSPZ vaccines.

**Figure 1:**
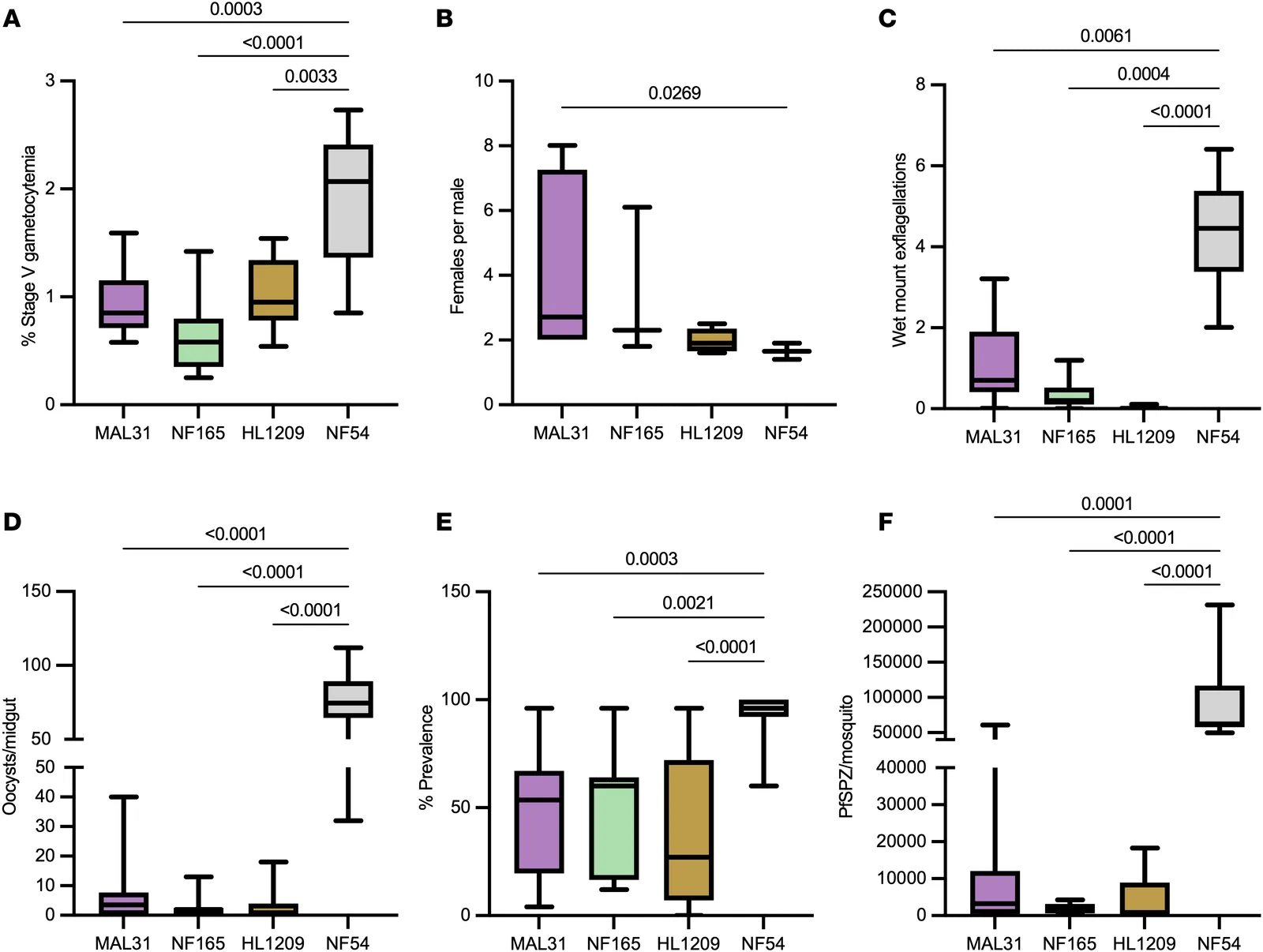
Gametocyte culture, gametogenesis, and mosquito-stage development of Pf MAL31, NF165, HL1209, and NF54. (**A**) The percentage of mature (stage V) gametocytes relative to uninfected RBCs, counted on Giemsa-stained slides at ×100 magnification. (**B**) The ratio of female to male stage V gametocytes prior to mosquito feeds. (**C**) The average number of exflagellations of the male gametes in 10 fields of a wet mount slide counted under phase contrast and ×40 magnification. (**D**) Mean number of oocysts per mosquito midgut from *n* = 25 Mercurochrome-stained, dissected midguts. (**E**) Prevalence of oocyst infection in mosquito midguts determined from *n* = 25 dissected mosquitoes. (**F**) Number of PfSPZ per salivary gland determined at the time of dissection. Box-and-whisker plots show the median (center line), interquartile range (box), and minimum and maximum values (whiskers). Statistical significance was determined using the Kruskal-Wallis test.

To develop optimized pan-African hybrids as PfSPZ vaccine substrates with an expectation of higher protection than with NF54 and with the same manufacturing efficiency, we first assessed the in vitro development of gametocytes and their subsequent progression through key parasite stages following mosquito infection for 3 recently isolated East African strains. These included 2 Malawian strains, MAL31 (32) and NF165 (33), and HL1209 from South Sudan (34). These recent African isolates all produced gametocytes, albeit at lower numbers than NF54 (Figure 1A). The ratio of female and male stage V gametocytes was variable even though they were grown in identical conditions, with NF54 consistently producing a 2:1 female to male stage V gametocyte ratio (Figure 1B) and showing robust male fertility based on measurement of male gamete exflagellation centers. In the East African strains, however, male gametocytes failed to efficiently exflagellate despite reaching maturity (Figure 1C). In contrast, the mean number of oocysts per midgut in mosquitoes fed stage V gametocytes from the East African isolates was significantly lower than that observed for NF54 (Figure 1D). Infection prevalence also varied substantially among isolates, ranging from approximately 25% to 60%, and was inconsistent across experiments (Figure 1E), resulting in low PfSPZ yields per mosquito (Figure 1F). We also characterized Pf strains isolated from Brazil, Cambodia, and Thailand (Supplemental Table 1; supplemental material available online with this article; https://doi.org/10.1172/jci.insight.207272DS1), which were deemed not amenable for cost-effective vaccine production, because they also exhibited deficiencies in exflagellation, oocyst yields, infection prevalence in mosquitoes, and PfSPZ yields per mosquito. Overall, across all isolates tested, similar characteristics were found relative to NF54: gametocyte development was less robust, male gametocytes did not exflagellate as efficiently, oocyst prevalence per mosquito was reduced, and PfSPZ yields per mosquito were significantly lower.

### Recent African field isolate PfSPZ have increased infectivity to human liver–chimeric mice.

**Figure 2:**
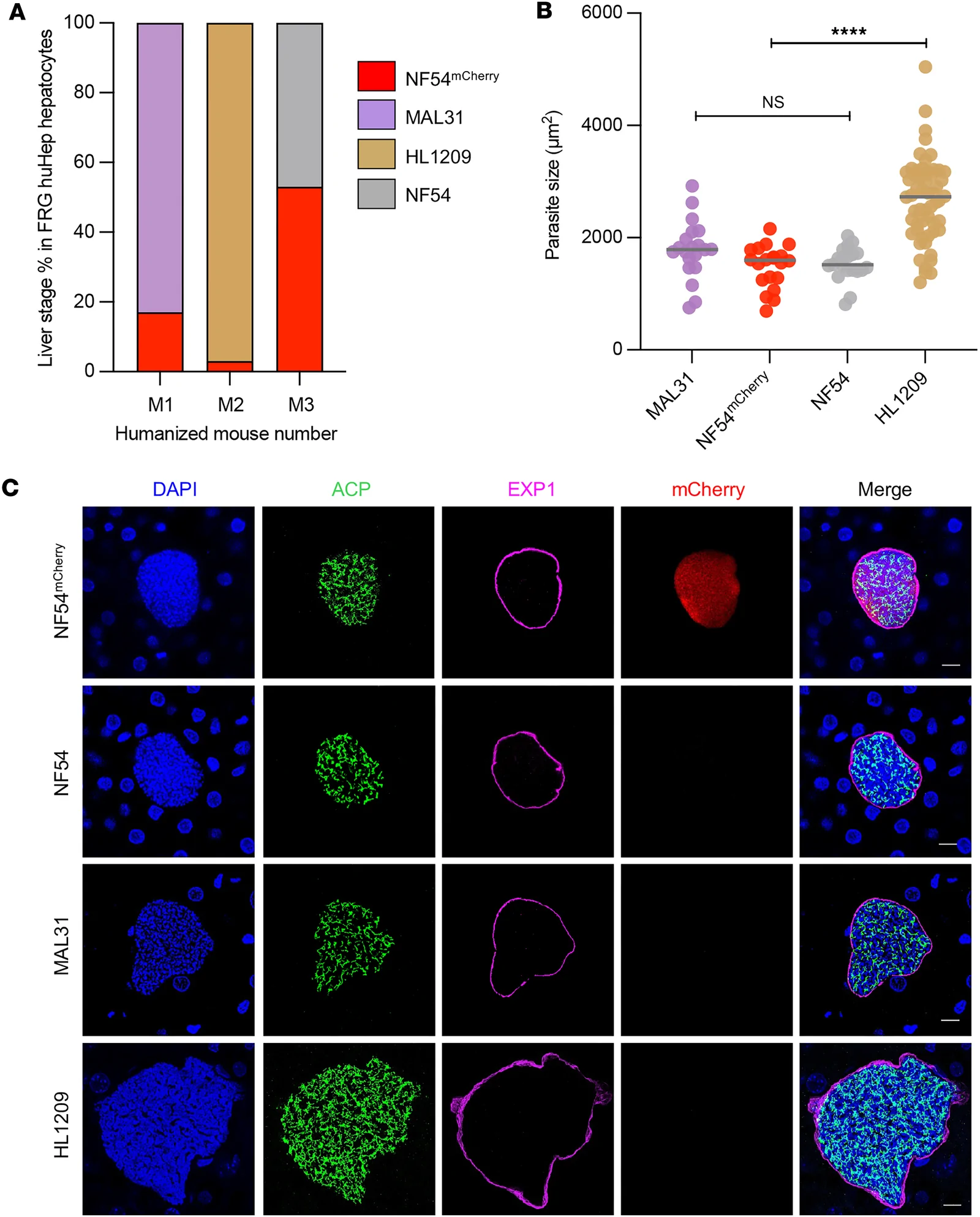
Liver-stage infectivity and growth of Pf NF54, MAL31, and HL1209 in the human liver–chimeric FRG NOD huHep mouse. (**A**) MAL31 and HL1209 sporozoites exhibit increased liver infectivity compared with NF54. Cryopreserved MAL31 or HL1209 sporozoites were co-inoculated with NF54^mCherry^ sporozoites, and liver-stage infection was assessed on day 6 by immunofluorescence analysis of at least 100 parasites. Individual FRG NOD huHep mice were injected with 2 × 10^6^ cryopreserved NF54^mCherry^ sporozoites (red) together with 2 × 10^6^ MAL31 (purple), HL1209 (gold), or NF54 (gray) sporozoites. Each column represents a single mouse (M1–M3), with colors indicating the proportion of parasites detected across 2 independent liver lobes (± SD). Absolute parasite counts are provided in Supplemental Table 2. (**B**) MAL31, NF54, and NF54^mCherry^ liver stages show similar sizes, whereas HL1209 liver stages are significantly larger. Each point represents an individual parasite (purple, red, and gray: *n* = 20; gold: *n* = 56); gray bars denote median values. Statistical significance was determined by 1-way ANOVA (****P < 0.0001). (**C**) Liver-stage parasites of MAL31 and HL1209 display nuclear morphology (DAPI, DNA, blue), apicoplast organization (ACP, green), and parasitophorous vacuole membrane expression (EXP1, magenta) comparable to NF54 and NF54^mCherry^ (mCherry, red). Day 6 liver sections (50 μm) were stained with antibodies against ACP, EXP1, and mCherry. Scale bars: 10 μm.

To assess liver-stage phenotypes of the recent East African parasite strains, we measured PfSPZ infectivity for the human liver. Previous research comparing PfSPZ infectivity in vitro in primary human hepatocytes demonstrated that PfSPZ from a Cambodian strain (NF135.C10) (18, 35) and a Nigerian strain (NF175) (19) were up to 8 times more infectious in vitro than NF54. To investigate the in vivo infectivity of the recently isolated East African strains for human hepatocytes and to compare their infectivity with that of NF54, we chose MAL31 and HL1209. We designed a coinfection experimental model, where individual human liver–chimeric FRG NOD huHep mice (36) were injected with 2 × 10^6^ purified, cryopreserved NF54^mCherry^ PfSPZ (37) (characterization of this transgenic line is presented in Supplemental Figure 1) and 2 × 10^6^ purified, cryopreserved MAL31 or HL1209 PfSPZ. Simultaneous infection of a single animal with 2 strains mitigates inter-mouse variability in infectivity rates, which is often driven by differences in human albumin levels reflecting the extent of liver humanization in FRG NOD mice (data not shown). At day 6 after infection, when MAL31 was compared with NF54, the mean percentage of liver-stage parasites was 83% MAL31 versus 17% NF54 (± SD 0.35%) (mean parasite numbers: MAL31 = 40.2 [± 11.3], NF54 = 8.4 [± 2.1]), demonstrating that MAL31 was approximately 5 times more infectious for the liver than NF54 (Figure 2A). For the HL1209 and NF54^mCherry^ coinfection, the difference was even more pronounced, with 97% of liver stages being HL1209 and only 3% NF54 (± 1.4%) (mean parasite numbers: HL1209 = 194.9 [± 29.7], NF54 = 4.5 [± 0.7]), more than a 30-fold difference (Figure 2A). To confirm that the NF54^mCherry^ transgenic parasites did not differ from wild-type NF54 in PfSPZ liver infectivity, we injected a mouse with 2 × 10^6^ purified, cryopreserved PfSPZ from both NF54 and NF54^mCherry^ lines. The 2 sporozoite populations were similarly infectious, with 47% NF54 and 53% NF54^mCherry^ (± 0.70%) (mean parasite numbers: NF54 = 15.5 [± 0.7], NF54^mCherry^ = 18 [± 1.4]) liver-stage parasites (Figure 2A). Parasite counts are detailed in Supplemental Table 2. Using immunofluorescence assay (IFA) to assess mature liver-stage morphology and size at day 6 of infection, we observed that all 4 parasite lines exhibited comparable morphology (representative images in Figure 2C), with well-branched apicoplasts and continuous parasitophorous vacuole membranes. In contrast, measurement of liver-stage size revealed that while NF54, NF54^mCherry^, and MAL31 parasites produced liver stages of similar dimensions, HL1209 liver stages were significantly larger (Figure 2B). Since increased numbers and size of late-liver-stage schizonts have been associated with enhanced immunogenicity and stronger T cell responses (38), this difference could have important implications for protective immunity. In summary, this result suggests that despite MAL31 and HL1209 having lower mosquito infectivity than NF54, they were significantly more efficient at establishing liver infections, with HL1209 generating liver stages 1.5-fold larger than NF54.

### Experimental genetic crosses between NF54 and recent African isolates create hybrid progeny with balanced contribution of parental genomes.

**Figure 3:**
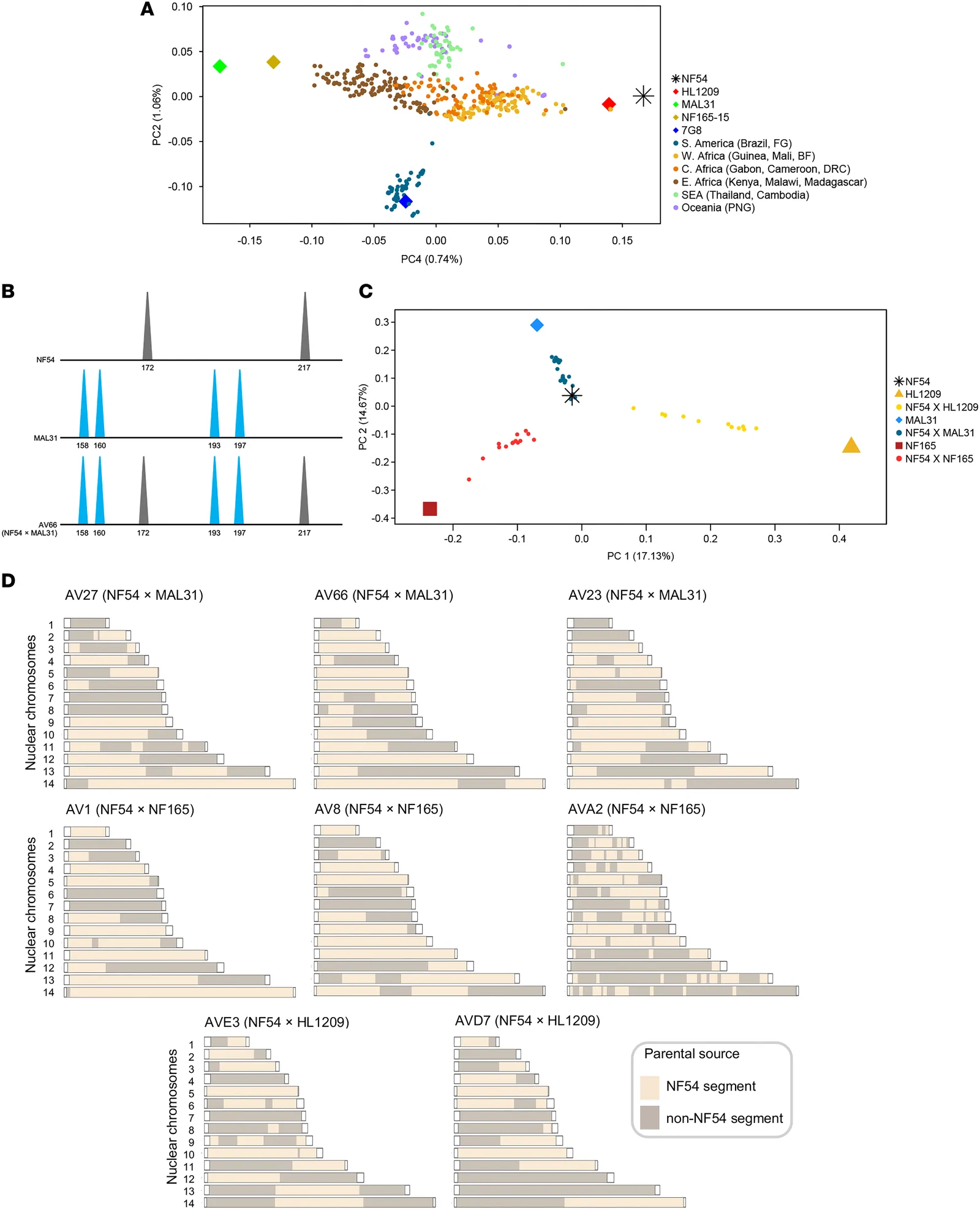
Genomic characterization of parental strains and hybrid progeny. (**A**) Genome-wide SNP-based PCA, using clinical isolates from several geographic regions (circles) and parental strains (larger symbols, diamonds and star). PC2 separates continents, while PC4 separates African isolates by longitude. MAL31 (green) and NF165 (gold) cluster with East African isolates (brown), while HL1209 (red) clusters with those from West Africa (yellow), including NF54 (star). (**B**) Graphical example of fragment analysis obtained through capillary electrophoresis for the NF54 × MAL31 cross. NF54 fingerprinting reveals 2 unique peaks (gray), and MAL31 parent has 4 unique peaks (blue). AV66, a potential recombinant clone, displays peaks derived from both parental lines. (**C**) PCA based on whole-genome sequence variants, including 4 parental strains (NF54, star; HL1209, triangle; MAL31, rhombus; NF165, square) and the hybrid clones (each dot represents 1 clone; different colors for each genetic cross). In each case, hybrid clones are intermediate in genetic composition relative to their respective parents. NF54 is shared by all hybrids and is centrally located. (**D**) Chromosome composition of 8 selected hybrid clones. Short-read, nonhuman whole-genome sequencing was used to infer chromosomal segments contributed by each parent. Contributions from NF54 are shown in beige and from the non-NF54 parental strain in gray. Subtelomeric regions derived from either parent (white) were excluded from the analysis. The unusually large number of inferred crossover events required to generate the genome structure observed in hybrid AVA2, derived from a cross between NF54 and NF165, suggests that this parasite line was not clonal.

In an effort to generate progeny with favorable mosquito and liver-stage phenotypes, while concurrently expanding antigenic diversity for downstream vaccine creation, we performed 3 separate genetic crosses between NF54 and MAL31, NF165, and HL1209. Mature stage V gametocytes were combined into a single infectious blood meal and fed to *A.* *stephensi* mosquitoes, which can result in the formation of hybrid ookinetes, and ultimately PfSPZ for life cycle continuation (Supplemental Table 3). PfSPZ populations from each cross were intravenously injected into FRG NOD huHep mice, and the mice were infused with human red blood cells (RBCs) to allow for the transition from liver-stage maturation to asexual blood-stage replication (39). Seven days after PfSPZ injection, infected blood was transferred to in vitro culture, and parasites were cloned for the isolation of hybrid progeny. We prescreened the genetic composition of the 117 potential hybrid clones that arose from the 3 genetic crosses using *RIFIN* fingerprinting (as an example, Figure 3B shows fingerprints for NF54 and HL1209). Based on the *RIFIN* fingerprinting, clones were deemed hybrid if they (a) displayed fingerprints from both parents or (b) presented a distinct fingerprint that did not match either parent (Supplemental Tables 4–6). Using this approach, we identified 31 of 60 potential hybrids from the NF54 × MAL31 genetic cross, 34 of 40 from the NF54 × NF165 genetic cross, and all 17 from the NF54 × HL1209 genetic cross. Of the 82 putative hybrid clones identified, 62 were selected for comprehensive genotyping, alongside 8 clones exhibiting parental fingerprints to validate the fingerprinting approach. De novo whole-genome assemblies were generated for each parental strain and differences identified relative to the reference *P.* *falciparum* 3D7, a clone of NF54 (15). MAL31 differed from 3D7 by 52.2 × 10^3^ single-nucleotide polymorphisms (SNPs) and 46.3 × 10^3^ indels distributed across the genome, including 17.4 × 10^3^ non-synonymous SNPs. HL1209 differed from 3D7 by 46.6 × 10^3^ SNPs and 45.6 × 10^3^ indels, including 16.1 × 10^3^ non-synonymous SNPs. As expected, NF54 differed from 3D7 by only 1.4 × 10^3^ SNPs, 8.4 × 10^3^ indels, and 80 non-synonymous SNPs (Supplemental Table 7). Principal component analysis (PCA) revealed that MAL31 and NF165 have genomic compositions consistent with their origin in East Africa, while HL1209, despite being isolated from a traveler to South Sudan, was more similar to isolates from West Africa (Figure 3A). To characterize the hybrids, we performed whole-genome sequencing of the 62 potential recombinants, which confirmed their identity (Supplemental Table 8). More than half of the hybrids exhibited balanced parental contributions — defined as ≥40% and ≤62% of the genome derived from the non-NF54 parent: 15 of 26 from NF54 × MAL31, 14 of 19 from NF54 × NF165, and 10 of 15 from NF54 × HL1209. As expected, PCA placed progeny genomes intermediate between their respective parents, exemplified in Figure 3C. These findings demonstrated that genetic crosses between East and West African strains are viable and established *RIFIN* fingerprinting as a prescreening tool that was a useful predictor of hybrid progeny (96% accuracy).

### The NF54 phenotype for high PfSPZ productivity is heritable in hybrid progeny.

**Figure 4:**
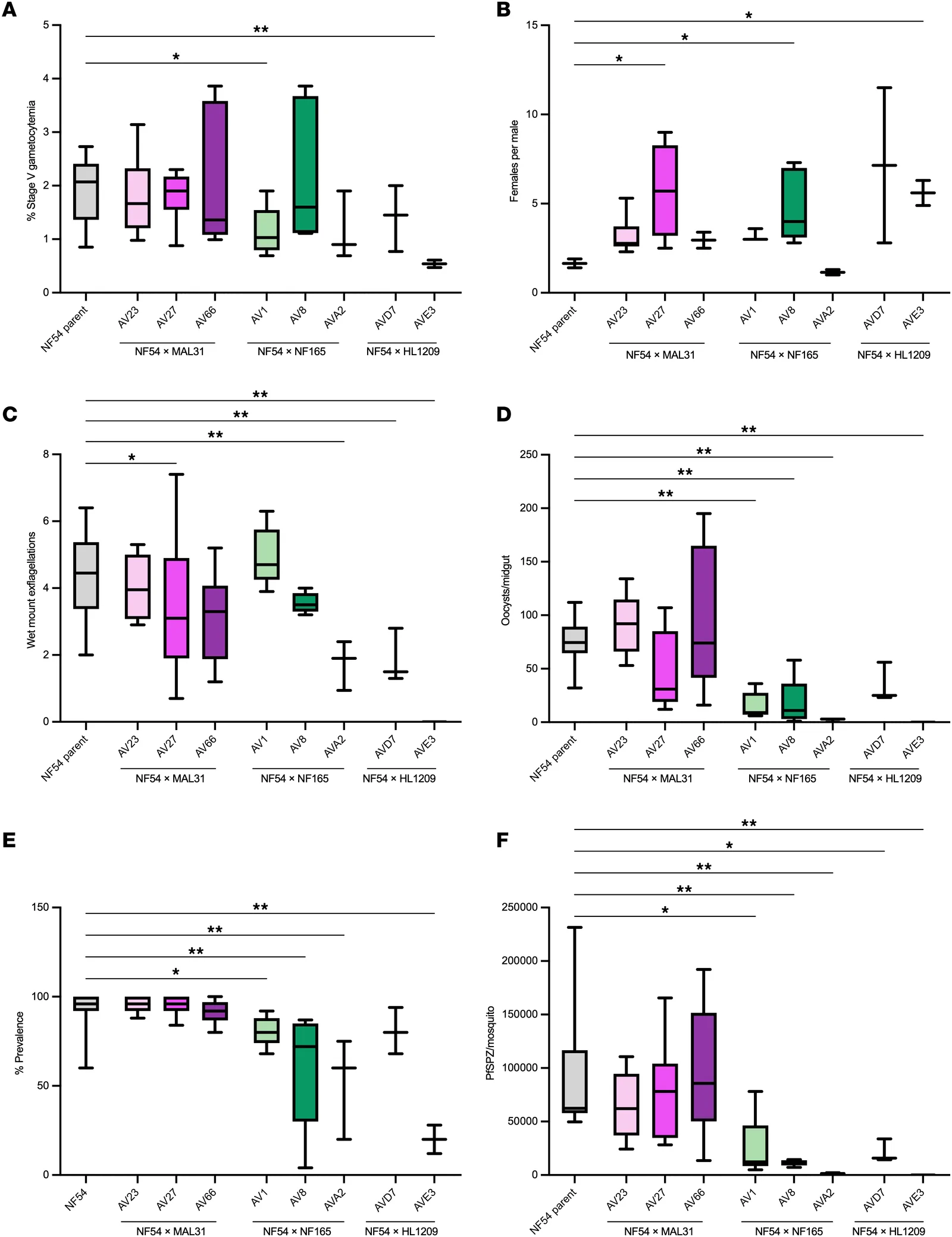
Gametocyte culture, gametogenesis, and mosquito-stage development of 8 recombinant parasites compared with NF54. (**A**) The percentage of mature (stage V) gametocytes relative to uninfected RBCs, counted on Giemsa-stained slides at ×100 magnification. (**B**) The ratio of female to male stage V gametocytes prior to mosquito feeds. (**C**) The average number of exflagellations of the male gametes in 10 fields of a wet mount slide counted under phase contrast and ×40 magnification. (**D**) Mean number of oocysts per mosquito midgut determined from *n* = 25 Mercurochrome-stained, dissected midguts. (**E**) Prevalence of oocyst infection among *n* = 25 mosquitoes assessed by midgut dissection. (**F**) The number of PfSPZ per salivary gland determined at the time of dissection. Box-and-whisker plots show the median (center line), interquartile range (box), and minimum and maximum values (whiskers). Statistical significance was determined using the Kruskal-Wallis test. *P < 0.05, **P < 0.01.

To identify hybrid progeny that potentially inherited the NF54 capacity to produce high numbers of PfSPZ in mosquitoes, we selected 8 hybrid clones with unique genome inheritance patterns (Figure 3D). Stage V gametocyte production was comparable to that of NF54 for the majority of clones (Figure 4A and Supplemental Table 9), with the exception of AV1 (NF54 × NF165) and AVE3 (NF54 × HL1209). Although female-to-male gametocyte ratios were higher than those seen with NF54 in some clones (Figure 4B), exflagellation was significant in all hybrids apart from AVE3 (Figure 4C). Consistent with this defect, AVE3 produced few oocysts and showed low oocyst prevalence, and no PfSPZ were detected in salivary glands (Figure 4, D–F). AV23, AV27, AV66 (NF54 × MAL31 progeny clones), and AVD7 (NF54 × HL1209) showed oocyst numbers (Figure 4D) and prevalence (Figure 4E) comparable to those seen with NF54, and the other hybrids did not. Interestingly, AV23, AV27, and AV66 produced similar numbers of PfSPZ to NF54 but AVD7 did not, despite high oocyst counts (Figure 4F and Supplemental Table 9). Further, mosquitoes infected with AV23 and AV66 displayed extensive oocyst melanization — absent in parental lines — with more than 90% of midguts affected (Supplemental Table 10). Moreover, in AV23, melanization led to substantial mosquito mortality, rendering the clone unsuitable for PfSPZ vaccine production (Supplemental Figure 2 and Supplemental Table 10). These results highlight the polygenic nature of gametocyte maturation and PfSPZ production and show that, among the hybrids tested, only AV27 and AV66 closely phenocopied NF54 across all measured mosquito-stage traits.

### The MAL31 phenotype for high infectivity of the FRG huHep mouse liver is heritable in hybrid progeny.

**Figure 5:**
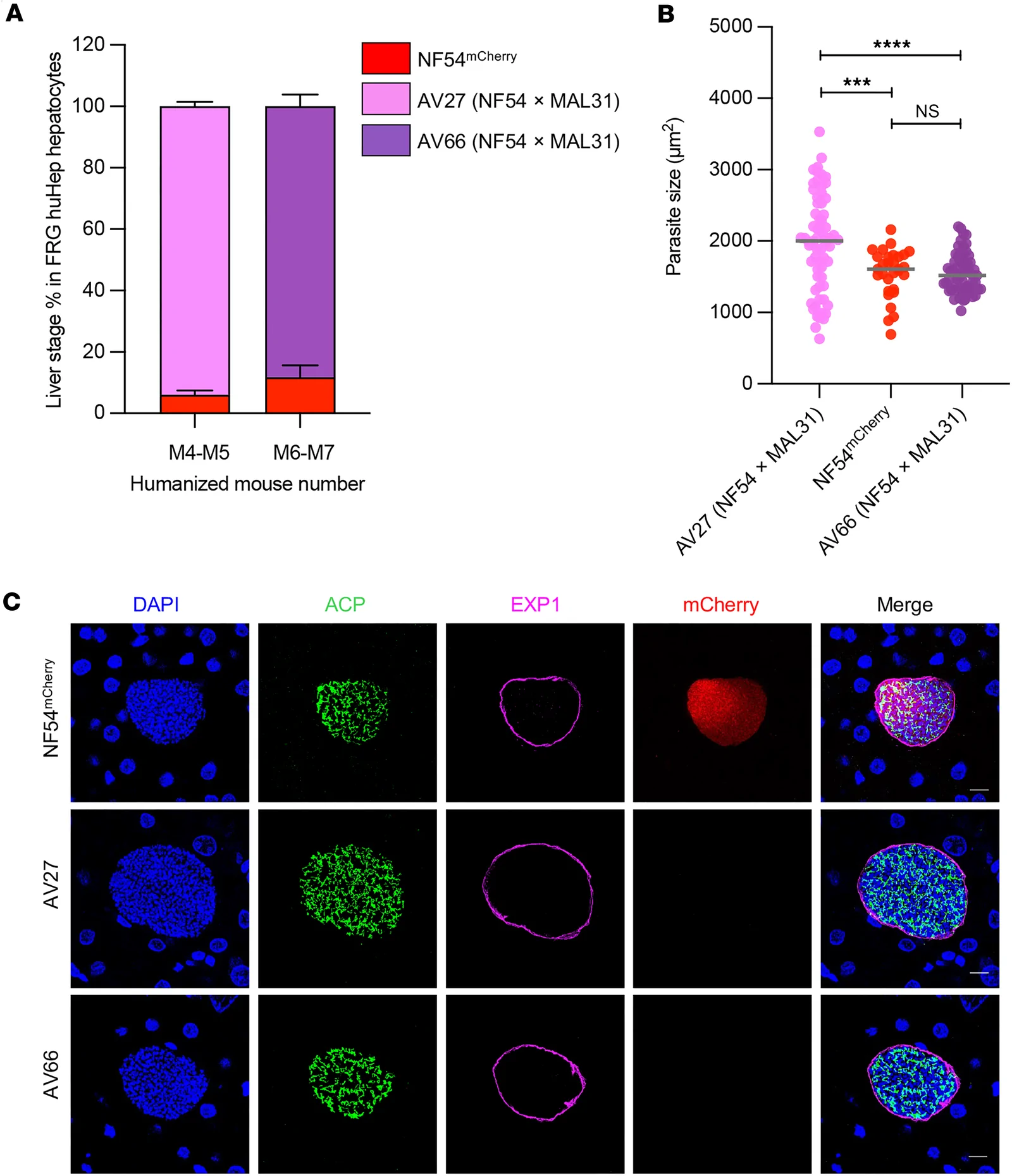
Recombinant AV27 and AV66 sporozoite progeny have increased infectivity for the FRG NOD huHep mouse liver compared with NF54^mCherry^ sporozoites. (**A**) AV27 (NF54 × MAL31) hybrid parasites are about 15-fold more infectious to human hepatocytes than NF54^mCherry^, whereas AV66 (NF54 × MAL31) hybrid shows approximately 7-fold higher infectivity. FRG NOD huHep mice were coinjected with 2 × 10^6^ cryopreserved NF54^mCherry^ (red) and 2 × 10^6^ AV27 (pink) or AV66 (violet) sporozoites. Coinfection experiments were performed in duplicate (M4–M5 for AV27; M6–M7 for AV66). Each color represents the mean percentage parasite burden (± SD) at day 6 after infection from 2 liver lobes of 2 mice as quantified by immunofluorescence assay. Absolute counts are provided in Supplemental Table 11. (**B**) Liver-stage parasites of AV66 (violet) and NF54^mCherry^ (red) exhibit similar sizes, whereas AV27 (pink) forms significantly larger liver stages than both AV66 and NF54^mCherry^. Each point represents a single parasite (AV27, *n* = 65; NF54^mCherry^, *n* = 30; AV66, *n* = 61). Gray bars denote median values. Statistical significance was assessed by 1-way ANOVA (***P = 0.0005; ****P < 0.0001). (**C**) Liver-stage parasites of AV27 and AV66 display comparable nuclear organization (DAPI, blue), apicoplast localization and expression (ACP, green), and parasitophorous vacuole membrane localization/expression (EXP1, magenta) to NF54^mCherry^ (mCherry, red). Day 6 liver sections (50 μm) were stained with antibodies against ACP, EXP1, and mCherry. Scale bars: 10 μm.

We used liver-stage infectivity and parasite size as surrogates for vaccine potency. To potentially identify hybrid parasites combining high PfSPZ yields with high liver-stage infectivity, we evaluated liver-stage infectivity in the 2 hybrids with the highest PfSPZ numbers without high mosquito mortality: AV27 and AV66 (NF54 × MAL31). We intravenously injected duplicate mice with 2 × 10^6^ purified, cryopreserved AV27 or AV66 PfSPZ and 2 × 10^6^ purified, cryopreserved NF54^mCherry^ PfSPZ and assayed infectivity at 6 days after infection. Compared with NF54, AV27 PfSPZ showed an approximately 15-fold increase in infectivity in FRG huHep mice, with 94% infectivity for AV27 versus 6% for NF54 (± 1.4%) (mean parasite numbers: mouse 4, AV27 = 94.8 [± 21.2] vs. NF54 = 6.9 [± 1.4]; mouse 5, AV27 = 262 [± 45.2] vs. NF54 = 14.4 [± 5.6]), while AV66 PfSPZ had an approximately 7-fold increase (Figure 5A and Supplemental Table 11), with 88% for AV66 versus 12% for NF54 (± 3.9%) (mean parasite numbers: mouse 6, AV66 = 84 [± 128] vs. NF54 = 8.7 [± 15.5]; mouse 7, AV66 = 109.4 [± 7.8] vs. NF54 = 18.8 [± 4.2]). For the AV27 liver infectivity study, FRG mice M4 and M5 were inoculated 1 month apart with PfSPZ derived from different production lots generated approximately 5 months apart, whereas mice M6 and M7 for AV66 were inoculated 4 months apart using the same SPZ lot. On day 6 after infection, AV66 liver-stage schizonts were comparable in size to NF54^mCherry^ liver-stage schizonts, whereas AV27 liver-stage schizonts were significantly larger than NF54 liver-stage schizonts (Figure 5B). Additionally, AV27 and AV66 liver stages were morphologically similar to NF54^mCherry^, based on expression patterns for DNA, ACP, and EXP1 (Figure 5C). Thus, our findings suggest that PfSPZ vaccines based on these hybrids would require substantially lower doses to elicit immune responses equivalent to those of NF54-based vaccines. This inference must be proven in a clinical trial.

### Phenotypic stability of hybrid clone AV27 in PfSPZ production and liver infectivity.

AV27 exhibited phenotypic stability across independent production cycles. NF54, the benchmark strain for PfSPZ yield, has maintained stable productivity over more than 20 years. Twelve production runs of NF54 from 2022 to 2024 yielded a median of 62,388 PfSPZ per mosquito (range 49,725–231,505). Under identical conditions, AV27 produced similar numbers (Supplemental Figure 3). Eleven production runs of AV27 from 2023 to 2025 yielded a median of 78,179 PfSPZ per mosquito (range 28,252–165,564) (NF54 vs. AV27, *P* = 0.5658, Mann-Whitney *U* test). In contrast, during the same period, MAL31 produced significantly lower numbers of PfSPZ than either NF54 or AV27 (Figure 4F and Supplemental Figure 3). Seventeen production runs of MAL31 from 2022 to 2024 yielded a median of 3,134 PfSPZ per mosquito (range 0–61,000) (NF54 vs. MAL31, *P* < 0.001, and AV27 vs. MAL31, *P* < 0.001) (Supplemental Figure 3). Liver-stage infectivity in the FRG huHep mouse was also consistent (Figure 5A) in 2 experiments in which the PfSPZ used for the independent infections were derived from independent production runs done about 5 months apart, with different sporozoite yields (34,700 and 86,400 PfSPZ per mosquito). Notably, the AV27 PfSPZ preparation used for the 2 different FRG huHep mouse infections was passaged 14 and 11 times, respectively, before induction of gametocytes, demonstrating durable phenotypic stability.

### Genomic characterization of the pan-African hybrid clone AV27.

Although both pan-African hybrid clones, AV27 and AV66, exhibited balanced genomic contributions from each parent and retained phenotypic traits supporting high PfSPZ production and robust in vivo hepatocyte infectivity — highlighting their suitability for PfSPZ-based vaccine development — AV27 emerged as the lead candidate owing to its markedly enhanced liver-stage infectivity as compared with AV66 (~15-fold higher than NF54 as compared with ~7-fold), its increased late-liver-stage parasite size, and its lack of melanization in the mosquito midgut. We also sought to identify a pan-African (East × West African) parasite with an expanded antigenic repertoire and thus undertook comprehensive genomic characterization of AV27, and CD8^+^ T cell epitope prediction analyses. We generated a complete genome assembly for AV27 (Table 1), showing that the hybrid differs from 3D7 (NF54 parent) by 27.3 × 10^3^ SNPs and from the MAL31 parent by 22.9 × 10^3^ SNPs. The assembly is ungapped, capturing the core and most subtelomeric regions of all nuclear chromosomes as well as both organellar genomes, within single contigs. At the chromosome level, about 50% of AV27’s genome was contributed by NF54 and about 50% by MAL31 (Supplemental Table 8).

### AV27 carries a robust representation of West and East African predicted CD8^+^ T cell epitopes.

PfSPZ vaccines are thought to protect primarily through CD8^+^ T cells that recognize 8– to 11–amino acid Pf peptides bound to class I HLA molecules on the surface of infected hepatocytes and then kill the infected hepatocyte (14). To determine whether AV27 had inherited similar numbers of potential CD8^+^ T cell epitopes from each parent, we used the core region of each chromosome to predict all strong-binding CD8^+^ T cell epitopes of 8–11 amino acids, recognized by 26 HLA alleles representative of the main allelic variants worldwide (40, 41) from NF54, MAL31, and AV27. Across all epitope lengths, NF54 had 2.2 million (1.15 million distinct, and the remainder duplicates) and MAL31 had 2.1 million (1.19 million distinct, and the remainder duplicates) predicted CD8^+^ T cell epitopes. Of all distinct predicted epitopes in each strain, 0.94 × 10^6^ (~80%) were shared by NF54 and MAL31 (Supplemental Tables 12–15). AV27 inherited 1.16 × 10^6^ (0.62 × 10^6^ distinct) from NF54 and 0.98 × 10^6^ (0.54 × 10^6^ distinct) from MAL31. Of all 1.16 × 10^6^ predicted distinct epitopes inherited from both parental strains, 0.11 × 10^6^ (9.5%) were unique to NF54, 0.12 × 10^6^ (10.3%) were unique to MAL31, and 0.93 × 10^6^ (80.2%) were present in both parental strains (predicted 9–amino acid epitopes are presented in Table 2, and predicted 8–, 10–, and 11–amino acid epitopes are provided in Supplemental Tables 16–18, respectively). Thus, NF54 and MAL31 share 80% of their predicted CD8^+^ T cell epitopes, and AV27 has inherited approximately equal numbers of unique epitopes from each parent. The identification of potential unique epitopes in AV27 from both East and West African strains supports the hypothesis that AV27 could confer broader vaccine protection across Africa than NF54.

## Discussion

To develop improved PfSPZ for vaccine development, we genetically crossed West African NF54, the current PfSPZ vaccine strain, with 3 Pf strains recently collected in East Africa, to obtain hybrid progeny. Hybrids were initially assessed by 3 criteria: (a) equivalent genomic representation of both parental strains, (b) robust production of PfSPZ in *A.* *stephensi* mosquitoes, and (c) increased liver-stage infectivity compared with NF54 (19). Of 82 candidates evaluated, 2 hybrid clones, AV27 and AV66, derived from a cross between NF54 and the East African isolate MAL31, met all these criteria. Ultimately, AV27 was selected as the lead candidate based on its liver-stage infectivity (~15-fold increase over NF54, compared with a 7-fold increase for AV66) and its larger late-liver-stage parasite size relative to NF54.

We posit that an AV27-based PfSPZ vaccine will be more efficacious overall in East and West Africa than current NF54-based vaccines for 3 reasons. First, infectivity of the liver is 15-fold higher, and we thus speculate that the same dose of PfSPZ vaccine will induce a greater magnitude of immune responses, which will lead to increased protection from challenge. Second, MAL31 was isolated in Malawi in 2016 (42) and is likely more representative of current circulating strains than NF54, which was isolated in 1979 (12). Third, we believe that any negative effect on VE in West Africa of reducing the West African immunopeptidome repertoire by about 10% will be overcome by the increased liver-stage infectivity and thus potency of AV27. Additionally, we hypothesize that protection in East Africa will be enhanced with the addition of the approximately 10% unique predicted epitopes from East Africa that are not present in NF54. Therefore, we predict that AV27-based PfSPZ vaccines will perform better than current NF54-based PfSPZ vaccines.

Our hypothesis that the increased liver infectivity of AV27 LARC2 PfSPZ will enhance liver-stage antigen presentation and improve protective efficacy awaits clinical evaluation. Nevertheless, prior PfSPZ-chemoprophylaxis vaccination (CVac) studies support a dose-dependent relationship with protection. In a PfSPZ-CVac (CQ) trial, in which participants received 3 doses of 3,200, 12,800, or 51,200 PfSPZ, protective efficacy against homologous controlled human malaria infection (CHMI) was 33% (3/9), 67% (6/9), and 100% (9/9), respectively, demonstrating increased protection with higher vaccine doses (4).

We further hypothesize that, based on predicted CD8^+^ T cell epitopes derived from both NF54 and MAL31 strains, the pan-African genetic composition of AV27 will confer broader protection against geographically diverse African parasites. Consistent with this rationale, prior studies have shown stronger protection against homologous versus heterologous CHMI (Pf7G8, a South American parasite that is genetically very distant from NF54) (16) following NF54 PfSPZ immunization. In one study, 5 doses of 270,000 PfSPZ protected 12 of 13 participants against homologous CHMI and 4 of 5 against heterologous CHMI 3 weeks after immunization; at 24 weeks, protection declined to 7 of 10 and 1 of 10, respectively. These findings suggest reduced durability of CD8^+^ T cell–mediated protection against genetically divergent parasites. Thus, collectively, these data support our hypothesis that AV27, by incorporating epitopes from both West African NF54 and East African MAL31 strains, may provide broader protection across Africa than NF54 alone.

In the field, the substantial genetic diversity of Pf (15) raises the same concerns. Sterile immunity targeting Pf liver stages is thought to rely primarily on long-lived tissue-resident CD8^+^ T cells that recognize CD8^+^ epitopes presented on the surface of infected hepatocytes in the context of class I HLA molecules (3, 14). The PfSPZ approach to vaccine development was undertaken in part because the thousands of antigens expressed by this complex eukaryotic pathogen result in presentation of many peptides presented on infected hepatocytes, providing diverse CD8^+^ epitopes to T cells (14, 43, 44). As long as some CD8^+^ epitopes are shared between the vaccine and the challenge Pf parasites, the summed protective responses can induce sterile immunity even against genetically distant strains of Pf. One of our goals was to create a hybrid Pf for vaccine development with increased numbers of potential CD8^+^ T cell epitopes for East African parasites. The immunopeptidome of AV27 has 80.2% of its predicted CD8^+^ T cell epitopes shared by NF54 and MAL31, 9.5% derived from NF54 alone, and 10.3% derived from MAL31 alone. AV27 thus includes about 90% of the immunopeptidome of each of its West and East African parental parasites, supporting our contention that we have been successful.

Phenotypic analyses of hybrid progeny revealed reproducible differences across biological replicates in sexual-stage development, mosquito-stage development, and liver-stage infectivity and growth, consistent with a strong genetic basis for these traits. For example, AV27 and AV66 from the NF54 × MAL31 cross consistently mirrored NF54 with respect to stage V gametocytemia levels, exflagellation of male gametes, mosquito midgut infection intensity and prevalence, and salivary gland PfSPZ per mosquito. These results would suggest that NF54-like phenotypes, if seen at the sexual stage, would predict downstream mosquito-stage outcomes. However, this was not always the case. Indeed, AV1 and AV8 from the NF54 × NF165 cross showed NF54-like exflagellation, although this did not lead to high oocyst counts and, ultimately, led to low PfSPZ per mosquito. Indeed, none of the NF54 × NF165 progeny yielded high PfSPZ counts, and this correlated with their low oocyst counts. Moreover, the AV27 (NF54 × MAL31) and AVD7 (NF54 × HL1209) hybrids exhibited comparable oocyst counts, both lower than those observed for NF54. However, only AV27 produced PfSPZ yields comparable to those of NF54, whereas AVD7 failed to reach similar levels. AV23 (NF54 × MAL31) produced oocyst burdens similar to those produced by NF54 but exhibited extensive oocyst melanization (>90% of midguts examined, compared with 0% for MAL31 and 8% for NF54) and increased mosquito mortality. Despite this pronounced effect, AV23 still generated PfSPZ numbers comparable to those seen with NF54. Melanization of midgut oocysts was also observed with AV66 (NF54 × MAL31); however, unlike AV23, this phenotype was not accompanied by increased mosquito mortality. Although melanization has been implicated in parasite-mosquito interactions (45), the determinants underlying its variable association with parasite development and mosquito survival remain unclear (46). Together, these findings demonstrate that hybrid progeny can inherit parental phenotypes while also exhibiting traits not observed in either parent, suggesting complex gene-gene interactions governing these observations. Nevertheless we acknowledge the relatively limited number of genetic crosses and hybrid clones we have assessed, which may not have captured the full phenotypic landscape.

The biological mechanisms underlying the uniquely high PfSPZ production of NF54 remain unclear. Likewise, the genetic and biological basis for the markedly enhanced liver-stage infectivity of recent field isolates, such as MAL31 and HL1209 — which are up to 30-fold more infectious to the human liver than NF54 — has yet to be defined. Our data demonstrate that these phenotypes are heritable. AV27 and AV66, the hybrid progeny of NF54 × MAL31, inherited the capacity for high PfSPZ production from NF54 and the enhanced liver-stage infectivity from MAL31. Further investigation, including quantitative trait locus mapping of multiple hybrid progeny displaying either high PfSPZ production or elevated hepatocyte infectivity, may identify the genetic determinants driving these phenotypes in both the mosquito stage and liver stage of the life cycle. Because genetic crosses are likely to enrich for the fittest progeny, confirmation of their relative fitness could enable the rational selection of field isolate–derived phenotypes for vaccine development. In addition, AV27 and AV66 are valuable parasites for studying Pf liver-stage development, a critical yet incompletely understood phase of the parasite life cycle.

Our current lead vaccine candidate is PfSPZ-LARC2 Vaccine, which contains 2 gene deletions (*PfMei2* and *PfLINUP*) (27). PfSPZ-LARC2 matures fully as a liver-stage schizont but does not produce infectious merozoites, resulting in a complete abrogation of the transition from liver stage to blood stage. Instead PfSPZ-LARC2 dies in situ, leading to local antigen presentation in the liver and liver-draining lymph nodes (27). A recent clinical trial in malaria-naive volunteers showed that, when administered by mosquito bite, a LARC parasite with a single gene deletion (*PfMei2*, known as GA2) provided greater VE to the EARD parasite, PfSPZ-GA1 (9). In a second clinical trial, GA2 induced 90% sterilizing protection against homologous CHMI after only 1 immunization by mosquito bite administration (10), suggesting that the injectable PfSPZ-LARC2 Vaccine will also be efficacious after a single dose. PfSPZ-LARC2 Vaccine is being assessed in Phase 1 clinical trials in Burkina Faso, the United States, and Germany (27) and in Phase 2 clinical trials in Burkina Faso and Mali. It has been shown to be fully attenuated, safe, and immunogenic. Protective efficacy results are expected in Q1 2027.

The methodology used in this study serves as a proof of concept, establishing the potential for future generations of hybrid parasite creation for advanced PfSPZ vaccines with equal representation of antigens from geographically and thus antigenically distinct parental strains. Moving forward, further hybridization could create vaccines for Southeast Asia, Oceania, and South America. Use of hybrid parasites for vaccine development is further supported by the fact that we found that the expression of an antigen repertoire spanning East and West Africa could be combined with key features promoting manufacturability and potency — high PfSPZ yield and substantially increased rates of hepatocyte infectivity by PfSPZ. Such a vaccine that promises as-yet unproven induction of high-level, sustained protection against Pf infection in sub-Saharan Africa that can prevent transmission of Pf and enable Pf elimination is urgently required. Overall, we have shown that we can generate hybrid progeny with high PfSPZ productivity in mosquitoes and high sporozoite infectivity for the liver and larger liver-stage parasites. Our contention that PfSPZ^HYB^-LARC2 (AV27-based) vaccine will offer improved protection at a lower dose and cost due to improved efficacy and breadth of immunogenic potential compared with PfSPZ-LARC2 (NF54-based) vaccine in sub-Saharan Africa remains to be demonstrated experimentally in human clinical trials.

## Methods

### Sex as a biological variable.

Our studies did not include humans. We did conduct studies in mice, specifically FRG KO huHep mice, which are immune-deficient mice (Fah^–/–^Rag2^–/–^Il2rg^–/–^) engrafted with human hepatocytes to create humanized livers. These immune-deficient mice with humanized livers are used because *Plasmodium falciparum* (Pf) sporozoites only invade and develop in human hepatocytes. The sex of the mice is irrelevant, because the mice themselves have no impact on the Pf infection of the human hepatocytes in these humanized livers and thus the sex of the mice has no relation to what might occur in male and female humans. (We have reported that in humans, males and females are protected at the same rate with whole-sporozoite PfSPZ vaccines [ref. 47].) We chose to use female FRG KO huHep mice because male FRG KO huHep mice are much more aggressive than females and, through fighting, often reduce the numbers of these expensive mice that can be successfully studied. Thus, sex of humans or mice was not a biological variable in our studies.

### In vitro cultivation of asexual Pf strains.

Pf laboratory strains (NF54, NF54^mCherry^), African field isolates (MAL31, NF165, HL1209), and the hybrid progeny clones generated from the experimental genetic crosses were cultured in complete growth medium (RPMI 1640 with l-glutamine and 25 mM HEPES supplemented with 9.1% [vol/vol] human serum and 33 μM or 367 μM hypoxanthine). Alternatively we used RPMI 1640 medium containing sodium bicarbonate and 4-(2-hydroxyethyl)-1-piperazineethanesulfonic acid (Invitrogen, Life Technologies), supplemented with 50 μM hypoxanthine (Sigma-Aldrich), 5% human serum O+ (BioIVT), and 5% AlbuMAX (Invitrogen, Life Technologies). Strains were cultured in O+ or A+ blood (BioIVT or Vitalant), at 2%–5% hematocrit in an atmosphere of 5% CO_2_, 5% O_2_, and 90% N_2_. Cell banks of Pf strains were generated in 50% glycerolyte and stored in liquid nitrogen vapor phase below –150°C.

### Gametocyte culture, assessment, and blood meal preparation.

Once asexual growth met criteria of greater than 1%–2% asexual parasitemia, gametocytogenesis was induced by stopping of the addition of fresh human RBCs to the culture and by changing of the medium daily until maturation. Gametocytes were cultured in either complete growth medium as detailed above, or sexual-culture medium composed of RPMI 1640 with sodium bicarbonate and 4-(2-hydroxyethyl)-1-piperazineethanesulfonic acid (Invitrogen, Life Technologies), supplemented with 50 μM hypoxanthine (Sigma-Aldrich) and 10% human serum (BioIVT). The cultures were maintained with O+ or A+ blood at 5% hematocrit.

On days 15–21 after induction, the cultures were assessed to measure percentage stage V gametocytes and percentage hematocrit to prepare a blood meal for feeding. Blood meals consisted of 0.1%–0.3% stage V gametocytes, fresh RBCs (40%–50% hematocrit), and human serum. A drop of approximately 20 μL of the resuspended blood meal was placed on a slide with a coverslip to count the wet mount exflagellation. The slide was incubated at 18°C for 20 minutes to induce exflagellation. After 20 minutes the slide was observed using a phase-contrast microscope (Olympus) to count exflagellation events at 10 positions of the coverslip using a ×40 objective. The female-to-male ratio of the stage V gametocytes was determined by counting of a total of 100 stage V gametocytes.

Hybrid cross blood meals were prepared as above for each strain and then mixed together with a 50%/50%, 75%/25%, or 90%/10% distribution, with higher ratios of the East African parent relative to NF54. For example, to create hybrids of MAL31 and NF54, the MAL31 parent strain consisted of 75% of the blood meal with the remaining 25% consisting of NF54. For each experiment, some of the blood meal from each parental strain was retained and fed to mosquitoes in addition to the hybrid feed.

### Growth, feeding, and maintenance of mosquitoes.

*Anopheles stephensi* (SDA500 strain) mosquitoes were reared in an environmental chamber (Harford) under 28°C ± 2°C, 75% relative humidity, and a 12-hour dark/12-hour light cycle. For colony maintenance and egg production, the females were fed with human whole blood (Vitalant) using artificial membrane feeders. Eggs were collected and seeded into pans to hatch, and larvae were maintained with fish food (Aqueon, Central Aquatics). Pupae were harvested on days 8–9 and transferred into cages, and after emergence, adult mosquitoes were maintained with water-soaked cotton balls and sugar cubes (Domino). Three- to 4-day-old *A.* *stephensi* females were heat-selected and 350–400 mosquitoes transferred into 1-gallon paper containers (Science Supplies WLE Corp.) for infectious feeding on day 5–6 post-emergence.

All operations with infected live mosquitoes were performed inside a secure, triple-screened insectary. Blood meals were offered in 3 to 5 mL feeders through an artificial membrane or parafilm and fed for 25–30 minutes using a circulating water bath set at 37°C. After feeding, the containers of mosquitoes were maintained in an incubator (Sanyo) at 26°C–27°C, 75%–80% humidity, and a 12-hour light/12-hour dark cycle inside the secure insectary. The mosquitoes were maintained with water-soaked cotton balls and sugar cubes or 8% dextrose solution in para-aminobenzoic acid water. Mosquitoes were fed a supplementary uninfected blood meal (a pre-warmed mixture of 50% human serum and 50% fresh human RBCs) at day 6–7 after infectious feed.

### Oocyst development and scoring.

On day 6–7 after infectious feed, 20–25 female mosquitoes out of the approximately 400 mosquitoes per gallon-sized container were transferred into a pint-sized container. The container was placed at –20°C for 10 minutes to anesthetize the mosquitoes. The midguts of Pf-infected females were dissected in 1× PBS, isolated, and stained with 0.2% Mercurochrome solution (Thermo Fisher Scientific) for 8 minutes. After staining, midguts were transferred to slides and covered with coverslips, and oocysts were scored under phase contrast at ×200. Data were compiled and analyzed using Graph Pad Prism (version 10.2.2).

### Midgut melanization assessment.

Mosquito midgut melanization (melanized oocysts or melanin vesicles) was assessed at 2 time points: during oocyst assessment (6–7 days after infection) and at salivary gland dissection for PfSPZ extraction (14–16 days after infection). Briefly, during oocyst assessment, 20 midguts were dissected on PBS and evaluated under a light microscope before Mercurochrome staining. Immediately after salivary gland dissection, the same number of midguts from dissected mosquitoes were assessed for melanization for each line. The prevalence was quantified by presence/absence of melanized midguts divided by total of assessed midguts. Mortality rates at the time of PfSPZ extraction were estimated by the dead mosquitoes in the bottom of the container.

### Salivary gland dissection and extraction, purification, and cryopreservation of sporozoites.

On days 16–18 after feeding, anesthetized mosquitoes were swirled in 70% ethanol and then transferred to Schneider’s Insect Medium (Sigma-Aldrich) or medium containing M199 with 1% human serum albumin. Salivary glands were dissected manually and dissociated using a GentleMACS dissociator (Miltenyi Biotec) to extract sporozoites or dissociated by grinding of salivary glands in Schneider’s Insect Medium. Purification of sporozoites from mosquito salivary gland material used a series of size-exclusion filters. The final concentration of sporozoites was 15 × 10^3^ sporozoites/20 mL in cryoprotectant, rate-frozen and stored at below –150°C in liquid nitrogen vapor phase (LNVP).

### Experimental FRG huHep NOD mice.

Female FRG NOD huHep mice (over 3 months old) were obtained from Yecuris Inc. Human hepatocyte repopulation was confirmed by measurement of human serum albumin levels, and only mice with serum albumin levels greater than 4 mg/mL, indicating more than 70% human hepatocyte repopulation, were used. Before the injection of Pf sporozoites, NTBC (which is used to maintain hepatocyte chimerism) was removed, and the mice were maintained for 7 days on drinking water containing 3% dextrose and were provided with wet food.

### Liver-to-blood stage transition of genetically crossed parasites in FRG humanized mice.

Two million cryopreserved SPZ from 3 independent genetic crosses (NF54 × MAL31, NF54 × NF165, and NF54 × HL1209) were intravenously injected into 3 FRG mice. On days 6 and 7 after infection, the humanized mice were given 400 μL of human RBCs (at 70% hematocrit). On day 7, 4 hours after the human RBC injection, FRG mice were exsanguinated, and the blood was washed 3 times with 10 mL of complete medium (RPMI 1640 with 25 mM HEPES, 2 mM l-glutamine, 50 μM hypoxanthine, 5% human serum, 5% AlbuMAX). Fresh human O+ blood was then added to the infected RBC pellet to cultivate the transitioned parasites in vitro at 2% hematocrit. Asexual blood-stage cultures were fed daily and maintained at 37°C in an atmosphere of 5% CO_2_, 5% O_2_, and 90% N_2_ (39).

### Pf hybrid parasite cloning by limiting dilution.

Once the percentage of the bulk transitioned parasites reached 0.3%, the Pf hybrid parasites were cloned by limiting dilution in 96-well flat-bottom plates. Cultures, containing single infected RBCs, were serially diluted and plated at a density of 0.5 parasites per well in 200 μL volumes at 2% hematocrit. The cultures were fed with RPMI complete medium containing 1% fresh human RBCs on days 7, 10, 12, and 14. On day 16, wells were screened for the presence of parasites by PCR amplification of the 18S rRNA gene using the following primers: 18S forward (5′-AACCTGGTTGATCCAGTAGTCATATG-3′) and 18S reverse (5′-CCAAAAATTGGCCTTGCATTGTTAT-3′). Positive wells were expanded, and genomic DNA was isolated from each of the 117 clones for analysis of the genomic composition of the hybrid progeny.

### Genomic composition of clonal progeny: prescreening through RIFIN fingerprinting.

The RIFIN multigene family was amplified using 2 pairs of fluorescein-tagged primers. Primer pair (a) consisted of forward, FAM 5′-TACGTTACATTATGTTTTA-3′, and reverse, FAM 5′-ATATGTATTGCGCTTTTA-3′, generating a 200 bp amplicon (48). Primer pair (b) included forward, FAM 5′-CCATTATATTAATATATTATTGTTTG-3′, and reverse, FAM 5′-TGTAGTTTTCATCCTTTC-3′, producing a 500 bp amplicon. The resulting amplicons were purified, quantified, and sent to Azenta Life Sciences for capillary electrophoresis. The analysis of the fragments was performed using the Microsatellite plug-in for Geneious Prime.

### Two-strain coinfection of a single FRG humanized mouse to evaluate liver-stage infectivity.

Two million cryopreserved SPZ of the control line (strain 1: NF54^mCherry^) and 2 million cryopreserved SPZ of the test line (strain 2: MAL31/HL1209/AV27/AVD7/NF54) were rapidly thawed, combined in a single syringe, and immediately injected intravenously into a humanized FRG mouse. The mice were sacrificed at 5 days and 18 hours (~6 days) after infection, and the liver was harvested and perfused with 1× PBS.

### Immunofluorescence assay.

Liver tissues were collected from Pf-infected FRG huHep mice 5 days and 18 hours after infection and fixed in 4% (vol/vol) paraformaldehyde (Alfa Aesar) prepared in 1× PBS. Fixed samples were sectioned at a thickness of 50 μm and blocked for 2 hours at room temperature with normal goat serum (1:500; Thermo Fisher Scientific). Sections were then washed twice with 1× PBS and incubated overnight at 4°C with the following primary antibodies: anti-mCherry (1:500; rat monoclonal, Invitrogen, M11217), anti-PfEXP1 (1:250; rabbit polyclonal, Antibody Facility, Walter and Eliza Hall Institute, Melbourne, Australia), and anti-ACP (Py ACP clone 40001, 1:500; mouse monoclonal, Stefan Kappe [University of Maryland, School of Medicine, Vice Chair, Translational Research, Department of Pediatrics; Director, Center for Vaccine Development and Global Health, Baltimore, USA]) (49). After primary antibody incubation, sections were washed twice in PBS and incubated for 2 hours at room temperature with appropriate secondary antibodies and DAPI. All antibody solutions were prepared in PBS containing 1% bovine serum albumin and 0.2% Triton X-100. After staining, sections were washed twice with 1× PBS, mounted in FluoroGuard anti-fade reagent (Bio-Rad), and stored at 4°C until imaging. Parasite quantification and size measurements were performed using a Keyence BZ-X700 fluorescence microscope and its associated analysis software. High-resolution images were acquired with a Stellaris 8 confocal microscope (Leica Microsystems), and post-acquisition deconvolution was carried out using Leica Lightning software. Image analysis was conducted using Fiji (ImageJ, NIH).

### Generation of whole-genome sequence data.

Genomic DNA was extracted from large-volume parasite cultures using the Monarch High Molecular Weight DNA Extraction Kit (New England Biolabs). Genomic DNA was extracted using a *Quick*-DNA 96 kit (Zymo Research). DNA samples were transferred to Maryland Genomics, at the Institute for Genome Sciences, for library construction and sequencing. Genomic libraries for sequencing on the Illumina NovaSeq 6000 were constructed using the NEBNext UltraExpress FS DNA Library Prep Kit (New England Biolabs). DNA was chemically fragmented, adapters ligated, and libraries amplified according to the manufacturer’s protocol. Libraries were size-selected and purified after PCR with SPRIselect Beads (Beckman Coulter Genomics). A Revvity GX Touch was used to assess libraries for size and quantity before sequencing. Libraries were multiplexed on a 150 bp paired-end Illumina NovaSeq 6000 run. For each sample, sequencing data were generated to a minimum of 75% breadth of coverage of the Pf3D7 genome with at least 5× depth of coverage. Genomic DNA samples used for construction of genomic libraries for sequencing on a Pacific Biosciences (PacBio) platform were processed as follows: DNA sample was sheared with g-TUBE (Covaris), at 15 kb setting. Sheared genomic DNA was prepared into a PacBio sequencing library using SMRTbell Express Template Prep Kit 2.0 (Pacific Biosciences) following the manufacturer’s instructions. After preparation, the library was size-selected on the BluePippin instrument (Sage Science) to remove library fragments smaller than 10 kb. Before sequencing, the library was bound to sequencing polymerase with Sequel II Binding kit 2.2, then multiplexed and sequenced with Sequel II Sequencing kit 2.0 and SMRT cell 8M on a Sequel II instrument (Pacific Biosciences). Different amounts of sequencing data were obtained for each sample, depending on the number of multiplexed samples per run.

### De novo genome assembly and variant detection from assembly comparison.

De novo genome assemblies for parental and hybrid strains were generated using hifiasm v0.16.1-r375 (using “disable purge duplication” but otherwise default options) to assemble the PacBio CCS reads (50). Because of the high amount of sequencing data generated, 2 sets of assemblies were built: (a) a randomly sampled set of reads consisting of 25% of all reads were used for assembly; and (b) the longest 25% of all reads were used for assembly. The second approach resulted in less fragmented assemblies and was used for downstream analyses. In the case of HL1209, a contig corresponding to the mitochondrial genome was missing from the assembly generated in (b) above and therefore was obtained from the assembly generated in (a). Assemblies were corrected with Illumina data using Pilon v1.24 with the following parameters: --fix bases --mindepth 5 --K 85 --minmq 0 --minqual 35 (51). For each assembly, the longest contig containing the sequence of each organellar genome (mitochondrion and apicoplast) was identified and trimmed to match the complete organellar genome. All redundant contigs or contigs smaller than 5 kb were removed from the final assembly. Pairwise alignments of genome assemblies were generated using the nucmer utility from the MUMmer package v3.1 (52). Alignments were filtered using delta-filter -r to map each reference to a position in the query genome assembly. SNPs and short indels were identified from the filtered alignments using MUMmer’s show-snps utility. Consecutive single-base indels were merged using a custom script. Variant calls were then converted into a VCF-like tabular format, and SNPs and indels were separated using VCFtools v0.1.15 (53).

### Variant identification from short-read data.

Reads were aligned to the 3D7 reference genome obtained from VEuPathDB (54), using Bowtie 2 v2.3.4.3 (55). The resulting BAM files were processed according to the Genome Analysis Toolkit Best Practices documentation, including sorting and base recalibration (56). Joint SNP calling was done using HaplotypeCaller. Diploid calls were allowed to account for heterozygous positions in polyclonal samples. Additional hard filtering was done to remove potential false positives using filter DP < 5 || QUAL < 50 || FS > 14∙5 || MQ < 20. In downstream analysis, only biallelic SNPs were used. When appropriate, and using custom scripts, the major allele was called at heterozygous sites if supported by at least 70% of reads; or else the heterozygous site was called as missing. Genomic sites were classified as intergenic, intronic, synonymous, and non-synonymous using SnpEFF v4.3t (57). Genotypes at sites in the core genome (as defined elsewhere) (58), at sites with known drug-resistant mutations, and at loci that encode parasite proteins that potentially target a protective immune response (as identified elsewhere) (59) were recovered using VCFtools v0.1.15 (53).

### SNP segments and parental contribution.

SNP statistics were recovered using bcftools stats v1.11 (60). Since NF54 is one of the parental strains for all hybrids, and since NF54 is nearly identical to the reference Pf3D7 at the genomic level (15), non-reference (or alternate [ALT]) genotypes were attributed to the non-NF54 parent. Hybrid clones were characterized based on the distribution of ALT genotypes. ALT genotypes were used to define chromosome segments of non-NF54 origin (i.e., chromosomal segments inherited from the non-NF54 parent), as follows: ALT genotypes (or SNPs) within 50 kb of each other (approximately 3 cM in Pf) were considered part of the same chromosomal segment of non-NF54 origin; a chromosomal segment contains at least 3 SNPs, and is defined by the coordinates of the first and last SNP in the segment. The minimum proportion of genome inherited from the non-NF54 parent was obtained from the sum of the length of all non-NF54 chromosomal segments divided by the length of the nuclear genome of Pf3D7. Hybrids were considered to have a genome with balanced contribution from both parents if the non-NF54 parent contributed between 40% and 60% of genome.

### Epitope prediction.

CD8^+^ T cell epitopes were predicted across target loci using NetMHCpan v4.1 (61) with default parameters. Only strong-binding epitopes were considered in downstream analysis. Epitope prediction was done using the 26 most common HLA alleles globally (40).

### Clustering.

Principal component analysis (PCA) was conducted with the SNPRelate package (62) in R. Whole-genome sequence data were used for parental strains, hybrid clones, publicly available clinical isolates, and combination of these sets. Datasets were restricted to biallelic SNPs in the core nuclear genome, with the major allele called for heterozygous positions.

### Statistics.

Statistical comparisons for gametocyte, oocyst, and PfSPZ assessments were performed by Kruskal-Wallis test with an uncorrected Dunn’s test for multiple comparisons using GraphPad Prism 10.2.2. The comparison between NF54 and AV27 passage number and PfSPZ yields was computed using Mann-Whitney *U* test. Statistical comparison between NF54 and NF54^mCherry^ sporozoites/mosquitoes was performed using 2-way ANOVA. For liver-stage infectivity estimation, standard deviations for the 2 parasite lines infecting FRG NOD mouse livers were calculated using parasite counts from each individual line measured in lobe 1 and lobe 2 of each mouse. Statistical significance for all liver-stage size measurements was determined using 1-way ANOVA with Tukey’s multiple-comparison test. A *P* value < 0.05 was considered significant. Measurements were taken from distinct samples.

### Study approval.

The study was performed in strict accordance with the recommendations in the *Guide for the Care and Use of Laboratory Animals* of the NIH, USA. To this end, the Seattle Children’s Research Institute (SCRI) has an Assurance from the Public Health Service (PHS) through the Office of Laboratory Animal Welfare (OLAW) for work approved by its Institutional Animal Care and Use Committee (IACUC). All of the work carried out in this study was specifically reviewed and approved by the SCRI IACUC.

### Data availability.

The parasite strains described in this article will be made available for the research community upon reasonable request and with appropriate material transfer agreements. All data used to generate the figures are provided in the Supporting Data Values XLS file. All whole-genome sequencing data generated are available from the NCBI’s Sequence Read Archive under BioProject PRJNA1235678, with BioSample accession numbers SAMN53535860–SAMN53535923 and SAMN53536485–SAMN53536494.

## Author contributions

BKLS, SLH, and AMV conceived the study. LP, BJ, AD, AP, YA, TVP, UR, PG, NR, CG, YY, SK, MK, GZ, JCS, AMV, and BKLS generated and analyzed data. LP, BJ, AD, AP, YA, TVP, PG, JCS, AMV, and BKLS developed methodology. LP, BJ, ML, SLH, JCS, AMV, and BKLS wrote the manuscript. JCS, AMV, and BKLS supervised the study. BKLS and AMV acquired funding.

## Conflict of interest

BJ, AP, YA, TVP, UR, SLH, and BKLS are employees of Sanaria Inc. BKLS and SLH own stock in Sanaria.

## Funding support

This work is the result of NIH funding, in whole or in part, and is subject to the NIH Public Access Policy. Through acceptance of this federal funding, the NIH has been given a right to make the work publicly available in PubMed Central.

- NIH grant U01 AI155356 (to BKLS).
- NIH grant P01 AI127388 (to AMV).

## Supplementary Material

Supplemental data

Supporting data values

## Figures and Tables

**Table 1:**
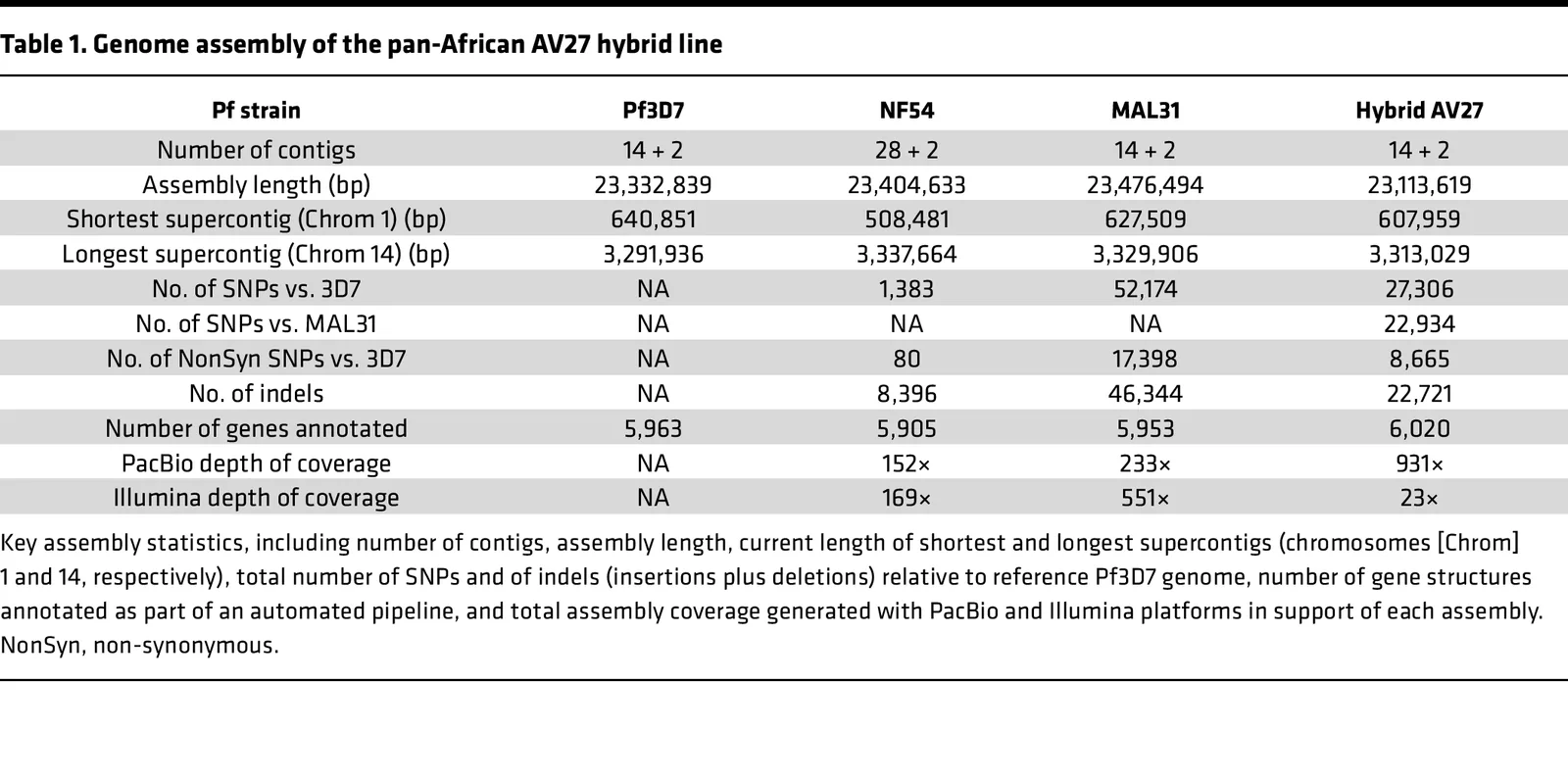
Genome assembly of the pan-African AV27 hybrid line

**Table 2:**
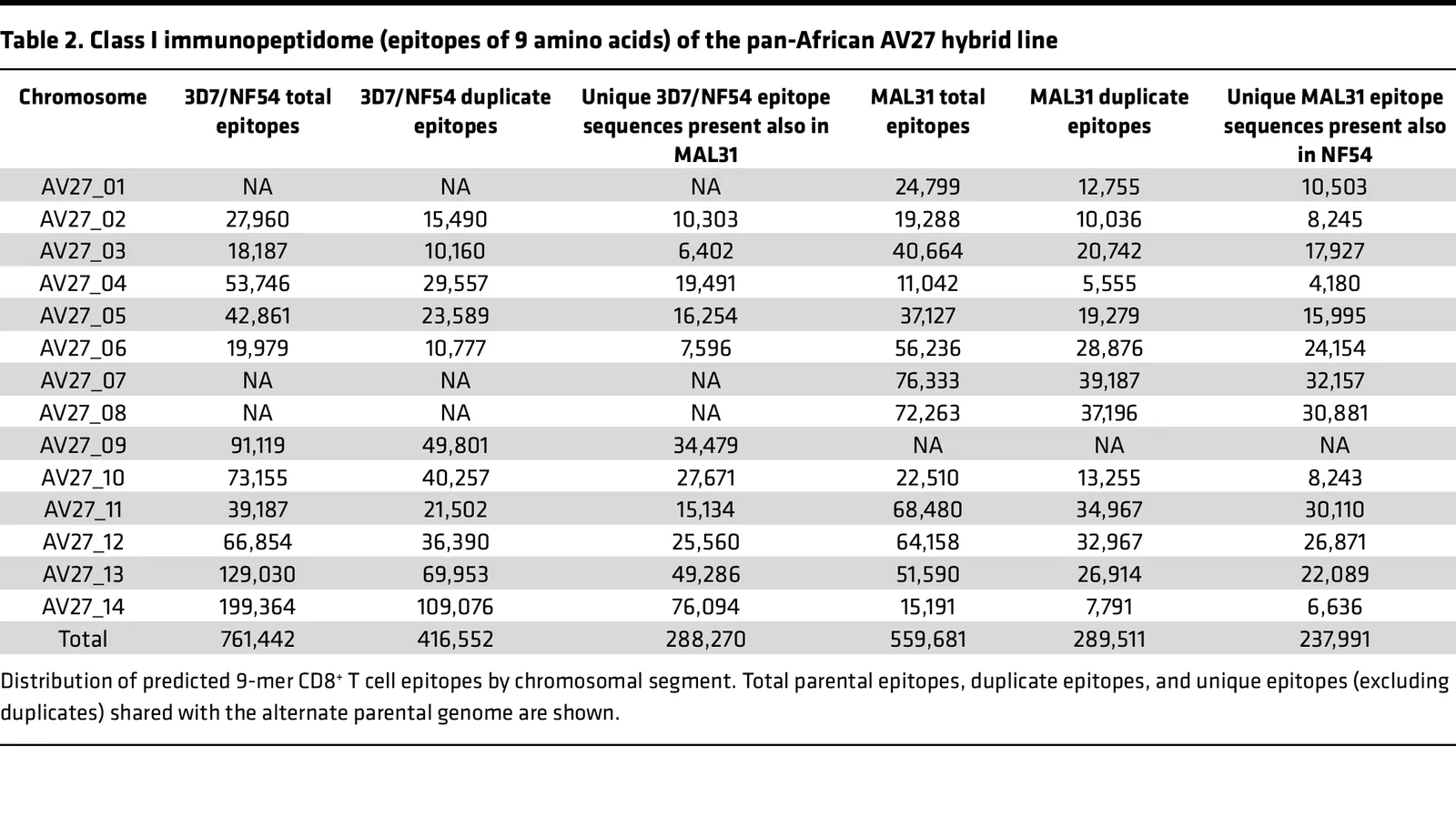
Class I immunopeptidome (epitopes of 9 amino acids) of the pan-African AV27 hybrid line

## Version 1 08/18/2026

In-Press Preview

## Version 2 09/22/2026

Electronic publication
