# Supplementary material for: Pan-African hybridization of PfSPZ increases antigenic diversity and replicative capacity for malaria vaccine design
Source: JCI Insight. 2026 Aug 18;11(18):e207272. doi: 10.1172/jci.insight.207272 (PMC13596721; doi:10.1172/jci.insight.207272)

Supplemental Table 1. Gametocyte culture, gametogenesis, and mosquito stage development of geographically dispersed *Plasmodium falciparum* isolates. Strains from East and West Africa, Southeast Asia and South America were cultured and fed to mosquitoes. Gametocyte development, oocyst counts, and PfSPZ yields were assessed. Median values are shown.

**Strain**<br>**Location**	**Number of feeds**	**% Stage V gametocytemia**	**Female:male ratio**	**Wet mount exflagellation in blood meal**	**Oocysts/mosquito**	**% Prevalence**	**PfSPZ/mosquito**<br>**(range)**
**NF54**<br>**W. Africa**	12	2.07	1.7	4.5	75	96	**62,388**<br>(49,725 – 231,505)
**MAL31 C11**<br>**Malawi**	17	0.85	2.7	0.7	3.5	54	**3,257**<br>(0 – 61,000)
**NF165**<br>**Malawi**	10	0.58	2.3	0.2	2	60	**2,050**<br>(182 – 4,245)
**CC102**<br>**Malawi**	3	0.29	ND	0.8	ND	ND	**334**<br>(0 – 819)
**HL1209**<br>**S. Sudan**	15	0.95	1.9	0	0	27	**551**<br>(0 – 18,287)
**M0736**<br>**Tanzania**	1	0.65	2	1.7	3	65	**2,626**
**NF166**<br>**Guinea**	15	0.81	ND	1.3	ND	ND	**5,604**<br>(0 – 19,839)
**Pf657-2**<br>**Cambodia**	10	1.07	6.1	0	10	60	**17,233**<br>(1,424 – 25,737)
**NHP4026**<br>**Thailand**	14	0.08	2.8	0	2	46	**4,792**<br>(0 – 17,217)
**NF135.C10**<br>**Cambodia**	13	1.23	ND	0	11	90	**19,980**<br>(6,192 – 88,320)
**7G8**<br>**Brazil**	19	1.43	ND	0.7	15	80	**11,474**<br>(2,012 – 42,600)

Supplemental Table 2. Absolute counts of MAL31, HL1209, NF54 and NF54^{mCherry} liver stage parasites at day 6 in FRG huHep mice, as detected by IFA staining.

Experiment	Section	r1 (mm)	r2 (mm)	Area (mm2)	ast African paren	NF54 mCherry	Total parasites	liver stages/cm2	East African / NF54 mCherry (%)
NF54 mCherry + MAL31 M1, Lobe 1	NL1.1	10	20	157	10	2	12	7	83/17
	NL2.1	10	20	157	13	1	14		
	NL2.2	10	22	173	8	2	10		
	NL3.1	11	22	190	8	1	9		
	NL3.2	11	20	173	10	4	14		
	**TOT**			**849**	**49**	**10**	**59**		

NF54 mCherry + MAL31 M1, Lobe 2	NL4.1	8	16	100	3	0	3	7	82.5/17.5
	NL4.2	7	15	82	9	2	11		
	NL4.3	9	15	106	7	2	9		
	NL4.4	6	15	71	7	1	8		
	NL5.1	5	14	55	2	1	3		
	NL5.2	7	16	88	3	0	3		
	NL5.3	5	16	63	2	1	3		
	**TOT**			**565**	**33**	**7**	**40**		

NF54 mCherry + HL1209 M2, Lobe 1	NL6.1	10	16	126	39	0	39	23	96/4
	NL6.2	11	15	130	18	1	19		
	NL6.3	11	15	130	29	2	31		
	NL7.1	12	16	151	29	0	29		
	NL7.2	11	16	138	37	1	38		
	NL7.3	10	15	118	23	1	24		
	**TOT**			**791**	**175**	**5**	**180**		

NF54 mCherry + HL1209 M2, Lobe 2	NL8.1	16	9	113	34	1	35	33	98/2
	NL8.2	15	4	47	31	1	32		
	NL9.1	17	9	120	24	1	25		
	NL9.2	14	5	55	34	0	34		
	NL10.1	17	7	93	29	1	30		
	NL10.2	16	9	113	27	0	27		
	NL10.3	18	9	127	38	0	38		
	**TOT**			**669**	**217**	**4**	**221**		

Experiment	Section	r1 (mm)	r2 (mm)	Area (mm2)	NF54	NF54 mCherry	Total parasites	liver stages/cm2	NF54 / NF54 mCherry (%)
NF54 mCherry + NF54 M3, Lobe 1	LN1.1	8	9	57	0	1	1	4	47/53
	LN1.2	9	10	71	0	1	1		
	LN1.3	4	6	19	2	0	2		
	LN1.4	5	9	35	0	1	1		
	LN2.1	10	10	79	1	1	2		
	LN2.2	6	11	52	1	2	3		
	LN2.3	5	9	35	1	0	1		
	LN5.1	7	5	27	0	0	0		
	LN5.2	9	7	49	1	0	1		
	LN5.3	10	8	63	0	0	0		
	LN5.4	9	6	42	0	0	0		
	LN6.1	12	7	66	4	3	7		
	LN6.2	7	4	22	0	0	0		
	LN6.3	8	9	57	0	1	1		
	LN6.4	9	5	35	0	1	1		
	LN7.1	11	6	52	1	2	3		
	LN7.2	9	6	42	1	2	3		
	LN7.3	6	6	28	2	1	3		
	LN7.4	9	5	35	1	1	2		
	**TOT**			**867**	**15**	**17**	**32**		

	Section	r1 (mm)	r2 (mm)	Area (mm2)	NF54	NF54 mCherry	Total parasites	liver stages/cm2	NF54 / NF54 mCherry (%)
NF54 mCherry + NF54 M3, Lobe 2	LN3.1	9	12	85	1	1	2	3	46/54
	LN3.2	6	11	52	0	0	0		
	LN3.3	9	14	99	2	1	3		
	LN3.4	7	10	55	1	2	3		
	LN4.1	10	15	118	1	2	3		
	LN4.2	8	9	57	1	1	2		
	LN4.3	6	6	28	1	1	2		
	LN8.1	8	11	69	1	3	4		
	LN8.2	12	13	122	1	1	2		
	LN8.3	7	12	66	1	1	2		
	LN8.4	8	10	63	2	2	4		
	LN8.5	10	11	86	1	1	2		
	LN9.1	9	13	92	2	1	3		
	LN9.2	7	10	55	1	1	2		
	LN9.3	9	14	99	0	1	1		
	**TOT**			**1145**	**16**	**19**	**35**		

Supplemental Table 3. Gametocyte, oocyst, and PfSPZ assessments of hybrid mosquito feeds. Grey highlights denote cryopreserved crosses that were transitioned through FRG mice to generate hybrid progeny.

	HL1209/NF54 Hybrid Cross					MAL31/NF54 Hybrid cross				NF165/NF54 Hybrid Cross				
	HL1209	Cross 1: 1:1	Cross 2: 3:1	Cross 3: 9:1	NF54	MAL31	Cross 1: 1:1	Cross 2: 3:1	NF54	NF165	Cross 1: 1:1	Cross 2: 3:1	Cross 3: 9:1	NF54
Stage V (%) in culture	1.34	N/A	N/A	N/A	2.13	0.4	N/A	N/A	0.85	0.76	N/A	N/A	N/A	2.14
Maturation day	17	N/A	N/A	N/A	18	17+20	N/A	N/A	18	17+20	N/A	N/A	N/A	17
Stage V concentration in the blood meal	0.1	0.1	0.1	0.1	0.1	0.11	0.1	0.11	0.11	0.1	0.1	0.1	0.1	0.1
Wet mount exflagellation of blood meal per field (under X40 phase contrast)	0	0.9	0.4	0.2	4.6	1.6	4.0	2.7	5.3	1.2	2.3	1.9	1.2	5.2
	Oocyst assessment					Oocyst assessment				Oocyst assessment				
Prevalence	35%	96%	90%	92%	100%	20	68	64	60	60	100	87	199	93
Oocysts/mosquito	1	57	51	15	79	1	62	42	70	2	23	21	17	91
	PfSPZ assessment					PfSPZ assessment				PfSPZ assessment				
PfSPZ/mosquito	1,116	48,471	55,200	30,417	166,219	187	66,446	14,885	57,704	3,832	46,117	25,945	12,686	119,221
Vials cryopreserved	0	19	23	16	0			8			15	10	3	

Supplemental Table 4. Genomic composition prediction of 60 NF54 × MAL31 clones through RIFIN fingerprinting. Fragment analysis of the parental lines NF54 and MAL31 show exclusive peaks, respectively grey and purple, identifying unique microsatellite profiles. Predicted genotypes can be: “P” parental, “R/B” recombinant showing peaks from both parents, “R/I” recombinant with peaks from one parent but not matching the parental complete profile. Left, parental controls and clones from AV10 to AV42; Right, parental controls and clones from AV43 to AV72.

Parasite ID	Peaks						Predicted genotype
NF54			172			217	P
MAL31	158	160		193	197		P
AV10	158	160	172			217	R/B
AV11			172			217	P
AV12		160	172			217	R/B
AV13		160				217	R/B
AV14	158	160		193	197		P
AV15	158	160	172	193	197		R/B
AV16	158	160		193	197		P
AV17			172			217	P
AV18			172			217	P
AV19	158	160	172	193	197		R/B
AV20			172			217	P
AV21	158	160	172	193	197	217	R/B
AV22	158	160	172			217	R/B
AV23	158	160	172	193	197		R/B
AV24	158	160		193	197		P
AV25	158	160	172	193		217	R/B
AV26	158	160	172	193	197	217	R/B
AV27	158	160	172				R/B
AV28		160	172	193	197		R/B
AV29	158	160	172	193	197	217	R/B
AV30	158	160		193	197		P
AV31	158	160		193	197		P
AV32		160	172				R/B
AV33	158	160		193	197		P
AV34			172			217	P
AV35			172			217	P
AV36	158	160	172				R/B
AV40			172			217	P
AV41			172			217	P
AV42		160	172				R/B

Parasite ID	Peaks						Predicted genotype
NF54			172			217	P
MAL31	158	160		193	197		P
AV43	158	160	172				R/B
AV44	158	160					R/I
AV45	158	160	172				R/B
AV46			172			217	P
AV47			172			217	P
AV48			172			217	P
AV49			172			217	P
AV50			172			217	P
AV51			172			217	P
AV52		160					R/I
AV53	158	160	172	193		217	R/B
AV54			172			217	P
AV55			172			217	P
AV56			172			217	P
AV57			172			217	P
AV58			172			217	P
AV59			172			217	P
AV60	158		172			217	R/B
AV61			172			217	P
AV62			172			217	P
AV63		160	172			217	R/B
AV64	158	160	172	193	197		R/B
AV65	158	160	172			217	R/B
AV66	158	160	172	193	197	217	R/B
AV67	158	160	172	193	197	217	R/B
AV68	158	160	172	193	197	217	R/B
AV69				193	197	217	R/B
AV70		160	172	193			R/B
AV71			172			217	P
AV72		160	172			217	R/B

Supplemental Table 5. Genomic composition prediction of 40 NF54 × NF165 clones based on RIFIN fingerprinting. Fragment analysis of the parental lines NF54 and NF165 revealed distinct, exclusive peaks—represented in grey and green, respectively—corresponding to unique genetic fingerprints. The upper panel shows the 200 bp fingerprints, characterized by a single NF165-specific peak, while the lower panel presents the 500 bp fingerprint, which displays additional unique peaks allowing clearer discrimination of NF165-derived profiles. Predicted genotypes for the cloned progeny (AV1–AVC13) are categorized as follows: “P” indicates parental type; “R/B” recombinant clones displaying peaks from both parents; and “R/I” recombinant clones exhibiting peaks from only one parent without fully matching the complete parental profile.

Parasite ID	Peaks			Predicted genotype
NF54		200	217	P
NF165	157			P
AV1	157	200		R/B
AV2		200		P
AV3		200		P
AV4	157		217	R/B
AV5	157	200		R/B
AV6	157	200		R/B
AV7	157	200		R/B
AV8	157	200	217	R/B
AV9		200	217	P

Parasite ID	Peaks									Predicted genotype
NF54	405								477	P
NF165		426	428	433	434	440	449	466		P
AVA1		426	428	433	434				477	R/B
AVA2							449			R/I
AVA3									477	R/I
AVA4		426	428	433	434			466		R/I
AVA5			428						477	R/B
AVA6			428	433			449	466	477	R/B
AVA7		426		433			449	466	477	R/B
AVA8			428	433	434		449	466		R/B
AVA9	405								477	P
AVB1			428	433			449	466		R/I
AVB2			428	433			449			R/I
AVB3			428	433			449	466	477	R/B
AVB4			428	433			449	466	477	R/B
AVB5		426		433				466	477	R/B
AVB6			428				449	466		R/I
AVB7			428	433			449	466	477	R/B
AVB8		426	428	433	434				477	R/B
AVB9			428	433				466	477	R/B
AVC1			428	433				466		R/I
AVC2							449	466	477	R/B
AVC3		426	428	433	434		449	466	477	R/B
AVC4		426	428	433	434			466	477	R/B
AVC5	405		428		434		449			R/B
AVC6		426		433			449		477	R/B
AVC7			428						477	R/B
AVC8			428		434		449			R/I
AVC9		426		433	434			466	477	R/B
AVC10		426	428	433				466	477	R/B
AVC11	405		428		434			466		R/B
AVC12		426		433			449			R/I
AVC13			428		434			466	477	R/B

Supplemental Table 6. Genomic composition prediction of 17 NF54 × HL1209 clones through RIFIN fingerprinting. Fragment analysis of the parental lines NF54 and HL1209 show exclusive peaks, respectively grey and yellow, identifying unique microsatellite profiles. “R/B” are recombinant clones displaying peaks from both parents while “R/I” recombinant clones exhibiting peaks from only one parent without fully matching the complete parental profile.

Parasite ID	Peaks														Predicted genotype
NF54		399	405	423		429		448		454		458		477	P
HL1209	387				426		433		450		456		469		P
AVD1					426		433		450	454	456		469	477	R/B
AVD2											456	458		477	R/B
AVD4		399		423	426	429	433	448		454			469	477	R/B
AVD7		399			426		433		450	454				477	R/B
AVD8		399			426		433		450	454	456			477	R/B
AVD9				423		429			450	454				477	R/B
AVE1	387										456			477	R/B
AVE2		399			426		433		450	454	456			477	R/B
AVE3				423	426	429	433				456		469		R/B
AVE4		399			426	429	433	448	450	454	456			477	R/B
AVE5				423		429			450	454	456			477	R/B
AVE6		399	405	423	426	429	433	448	450			458			R/B
AVE7				423	426	429	433		450	454	456			477	R/B
AVE8				423		429			450		456	458		477	R/B
AVE9		399		423		429		448		454		458		477	R/I
AVF1		399		423		429	433	448	450	454				477	R/B
AVF2		399		423	426	429	433	448		454					R/B

Supplemental Table 7. Genomic characterization of parental strains. Key assembly statistics, including number of contigs (nuclear chromosomes or shorter contigs plus the two organellar chromosomes), assembly length (cumulative length of contigs), current length of shortest and longest supercontigs (respectively, chromosomes 1 and 14), total number of SNPs and total number of indels (insertions plus deletions) relative to reference Pf3D7 genome, the number of gene structures annotated as part of an automated pipeline and the total assembly coverage generated with PacBio and Illumina platforms in support of each assembly. NF54 values were obtained from Moser et al. 2020.

	Pf3D7	NF54	MAL31	HL1209
Number of contigs	14 + 2	28 + 2	14 + 2	14 + 2
Assembly length (bp)	23,332,839	23,404,633	23,476,494	22,974,973
Shortest supercontig [Chrom 1] (bp)	640,851	508,481	627,509	664,780
Longest supercontig [Chrom 14] (bp)	3,291,936	3,337,664	3,329,906	3,217,930
# of SNPs	n/a	1,383	52,174	46,607
# of NonSyn SNPs	n/a	80	17,398	16,115
# of indels	n/a	8,396	46,344	45,560
Number of genes annotated	5,963	5,905	5,953	6,534
PacBio depth of coverage	n/a	152X	233X	865X
Illumina depth of coverage	n/a	169X	551X	117X

Supplemental Table 8. Genomic composition of 70 clones and matching with RIFIN fingerprinting prediction. Each cross is marked with a different pastel color. Clones phenotypically analyzed in the manuscript are shown in red. Clones with balanced genome contribution from both parents are highlighted in light blue, while clones with a single parental core genome are marked in grey.

	SNP Stats			Genome from non-NF54 parental			
Hybrid ID	NF54 sites	Non-NF54 sites	Missing genotypes	Cumulative length of segments (bp)	Genome proportion (%)	RIFIN fingerprinting prediction	Genome sequence and RIFIN fingerprinting matching
NF54 x HL1209: AVD1	24224	8793	7836	14,262,879	61.2	Recombinant	YES
NF54 x HL1209: AVD2	23617	2781	14455	8,957,848	38.5	Recombinant	YES
NF54 x HL1209: AVD3	25213	3612	12028	10,313,888	44.3	Recombinant	YES
NF54 x HL1209: AVD4	22509	6204	12140	14,854,019	63.8	Recombinant	YES
NF54 x HL1209: AVD7	26107	7890	6856	13,363,277	57.4	Recombinant	YES
NF54 x HL1209: AVD8	23798	5495	11560	13,036,993	56.0	Recombinant	YES
NF54 x HL1209: AVE1	21808	5594	13451	14,000,743	60.1	Recombinant	YES
NF54 x HL1209: AVE3	25920	6591	8342	11,866,648	50.9	Recombinant	YES
NF54 x HL1209: AVE4	25626	7162	8065	13,144,499	56.4	Recombinant	YES
NF54 x HL1209: AVE5	28918	3616	8319	12,386,977	53.2	Recombinant	YES
NF54 x HL1209: AVE6	30963	3106	6784	6,651,186	28.6	Recombinant	YES
NF54 x HL1209: AVE7	24397	7369	9087	14,300,291	61.4	Recombinant	YES
NF54 x HL1209: AVE8	29924	6081	4848	10,055,637	43.2	Recombinant	YES
NF54 x HL1209: AVE9	36085	17	4751	Not hybrid or subtelomeric recomb.		Recombinant	YES
NF54 x HL1209: AVF1	29730	5998	5125	9,492,977	40.8	Recombinant	YES
NF54 x HL1209: AVF2	28344	6188	6321	10,336,407	44.4	Recombinant	YES
NF54 x MAL31: AV10	31509	7	9337	Not hybrid or subtelomeric recomb.		Recombinant	YES
NF54 x MAL31: AV13	24177	14	16662	Not hybrid or subtelomeric recomb.		Recombinant	YES
NF54 x MAL31: AV15	19870	2279	18704	9,043,761	38.8	Recombinant	YES
NF54 x MAL31: AV16	22282	4406	14165	12,430,583	53.4	Parental	YES
NF54 x MAL31: AV19	36182	321	4350	2,615,933	11.2	Recombinant	YES
NF54 x MAL31: AV21	24290	11	16552	Not hybrid or subtelomeric recomb.		Recombinant	YES
NF54 x MAL31: AV22	27070	6326	7457	13,250,687	56.9	Recombinant	YES
NF54 x MAL31: AV23	27143	6739	6971	9,479,464	40.7	Recombinant	YES
NF54 x MAL31: AV24	29388	5381	6084	10,371,255	44.5	Parental	NO
NF54 x MAL31: AV25	33764	3691	3398	6,987,856	30.0	Recombinant	YES
NF54 x MAL31: AV26	31607	2419	6827	12,044,490	51.7	Recombinant	YES
NF54 x MAL31: AV27	26461	6729	7663	10,868,207	46.7	Recombinant	YES
NF54 x MAL31: AV28	29444	4398	7011	9,269,022	39.8	Recombinant	YES
NF54 x MAL31: AV29	30199	4049	6605	7,262,283	31.2	Recombinant	YES
NF54 x MAL31: AV31	20733	10025	10095	12,160,133	52.2	Parental	NO
NF54 x MAL31: AV32	39325	43	1485	Not hybrid or subtelomeric recomb.		Recombinant	YES
NF54 x MAL31: AV33	29709	5033	6111	9,857,873	42.3	Parental	NO
NF54 x MAL31: AV36	29838	5279	5736	9,381,253	40.3	Recombinant	YES
NF54 x MAL31: AV42	39362	26	1465	Not hybrid or subtelomeric recomb.		Recombinant	YES
NF54 x MAL31: AV43	36253	21	4579	Not hybrid or subtelomeric recomb.		Recombinant	YES
NF54 x MAL31: AV44	21147	3497	16209	11,985,735	51.5	Recombinant	YES
NF54 x MAL31: AV45	10932	893	29028	7,007,411	30.1	Recombinant	YES
NF54 x MAL31: AV46	34612	12	6229	21,497	0.1	Parental	YES
NF54 x MAL31: AV53	17958	2842	20053	12,665,589	54.4	Recombinant	YES
NF54 x MAL31: AV60	20073	2104	18676	9,873,000	42.4	Recombinant	YES
NF54 x MAL31: AV61	38523	90	2240	114,602	0.5	Parental	YES
NF54 x MAL31: AV63	16328	14	24511	Not hybrid or subtelomeric recomb.		Recombinant	YES
NF54 x MAL31: AV64	28145	3010	9698	7,273,255	31.2	Recombinant	YES
NF54 x MAL31: AV65	30132	6420	4301	9,711,954	41.7	Recombinant	YES
NF54 x MAL31: AV66	30658	4904	5291	8,618,818	37.0	Recombinant	YES
NF54 x MAL31: AV67	26783	2873	11197	7,224,870	31.0	Recombinant	YES
NF54 x MAL31: AV68	16208	2385	22260	12,106,166	52.0	Recombinant	YES
NF54 x MAL31: AV70	22889	4671	13293	12,333,063	52.9	Recombinant	YES
NF54 x NF165: AV1	24627	5551	10675	8,468,721	36.4	Recombinant	YES
NF54 x NF165: AV2	36163	51	4639	29,542	0.1	Parental	YES
NF54 x NF165: AV4	22799	10524	7530	14,891,814	63.9	Recombinant	YES
NF54 x NF165: AV7	25503	8620	6730	10,367,766	44.5	Recombinant	YES
NF54 x NF165: AV8	26739	6618	7496	8,745,584	37.5	Recombinant	YES
NF54 x NF165: AVA1	29280	5806	5767	10,733,020	46.1	Recombinant	YES
NF54 x NF165: AVA2	27823	4262	8768	12,875,387	55.3	Recombinant	YES
NF54 x NF165: AVA5	39200	45	1608	Not hybrid or subtelomeric recomb.		Recombinant	YES
NF54 x NF165: AVA6	29857	6085	4911	9,571,205	41.1	Recombinant	YES
NF54 x NF165: AVA7	27595	6188	7070	10,363,020	44.5	Recombinant	YES
NF54 x NF165: AVA8	29989	6234	4630	9,662,623	41.5	Recombinant	YES
NF54 x NF165: AVA9	37173	42	3638	Not hybrid or subtelomeric recomb.		Parental	YES
NF54 x NF165: AVB3	28024	6864	5965	11,506,540	49.4	Recombinant	YES
NF54 x NF165: AVB4	29906	6120	4827	9,575,929	41.1	Recombinant	YES
NF54 x NF165: AVB5	22699	8365	9789	16,479,519	70.8	Recombinant	YES
NF54 x NF165: AVB7	29104	5063	6686	9,519,064	40.9	Recombinant	YES
NF54 x NF165: AVB8	25618	6494	8741	13,072,149	56.1	Recombinant	YES
NF54 x NF165: AVB9	24893	5102	10858	9,779,208	42.0	Recombinant	YES
NF54 x NF165: AVC2	29599	4411	6843	9,867,532	42.4	Recombinant	YES
NF54 x NF165: AVC3	27936	6178	6739	12,332,663	52.9	Recombinant	YES
NF54 x NF165: AVC4	18253	3011	19589	12,557,635	53.9	Recombinant	YES
							**RIFIN prediction precision: 67/70 = 96 %**

Supplemental Table 9. Gametocyte culture, gametogenesis, and mosquito stage development of the 8 selected hybrid clones. Median values are shown.

Hybrid	Number of feeds	% Stage V Gametocytemia	Exflagellation	Female:male ratio	Oocysts / midgut	% Prevalence	PfSPZ / mosquito
**AV23**	10	1.665	3.95	2.775	92	96	62,147
**AV27**	10	1.915	3.15	5.3	29	96	69,696
**AV66**	6	1.36	3.3	2.95	74	92	85,695
**AV1**	5	1.19	4.7	3	9	80	12,311
**AV8**	5	0.84	3.5	4	11	72	11,689
**AVA2**	3	0.9	1.9	1.15	3	60	1,404
**AVD7**	3	1.45	1.5	7.15	25	80	15,873
**AVE3**	2	0.54	0	5.6	0	20	0

Supplemental Table 10. Summary of the melanization levels and mortality rate assessed at 14-16 dpi for *P. falciparum* parental strains and hybrid lines. Melanization incident was evaluated by presence/absence in 20 (n) dissected midguts. Mortality rate represents the number of dead mosquitoes assessed by eye in the bottom of the containers at the time of PfSPZ dissection.

**Pf Parental lines**	**Melanization prevalence**	**Mortality rate**
**NF54**	Exp #1: 2 out of 20 (10%)	Exp #1: 20%
	Exp #2: 1 out of 20 (5%)	Exp #2: 20%
	Exp #3: 2 out of 20 (10%)	Exp #3: 20%
	**Average:** 8.33%	**Average:** 20%
**MAL31**	Exp #1: 0 out of 20 (0%)	Exp #1: ≤ 20%
	Exp #2: 0 out of 20 (0%)	Exp #2: ≤ 20%
	Exp #3: 0 out of 20 (0%)	Exp #3: ≤ 20%
	**Average:** 0 out of 20 (0%)	**Average:** ≤ 20%
**Pf hybrid lines**	**Melanization prevalence**	**Mortality rate**
**AV27** (NF54 x MAL31)	Exp. #1: 0 out of 20 (0%)	Exp #1: ≤ 20%
	Exp. #2: 0 out of 20 (0%)	Exp #2: ≤ 20%
	Exp #3: 0 out of 20 (0%)	Exp #3: ≤ 20%
	**Average:** 0%	**Average:** ≤ 20%
**AV66** (NF54 x MAL31)	Exp #1: 18 out of 20 (90%)	Exp #1: 20%
	Exp #2: 19 out of 20 (95%)	Exp #2: 25%
	Exp #3: 18 out of 20 (90%)	Exp #3: 25%
	**Average:** 91.67%	**Average:** 23.33%
**AV23** (NF54 x MAL31)	Exp. #2: 18 out of 20 (90%)	Exp #2: 80%
	Exp. #3: 18 out of 20 (90%)	Exp # 3: 61%
	Exp #4: 19 out of 20 (95%)	Exp #4: 81%
	**Average:** 91.67%	**Average:** 74%

Supplemental Table 11. Absolute counts of AV27 and AV66 pan-African liver stage parasites at day 6 in FRG huHep mice, as detected by IFA staining.

Pan-African Hybrid liver stage infectivity in FRG huHep mice (day 6 post infection)											
**Experiment**	**Section**	**r1 (mm)**	**r2 (mm)**	**Area (mm2)**	**Hybrid**	**NF54 mCherry**	**Total parasites**	**liver stages/cm2**	**(%) hybrid / NF54 mCherry**	**Two FRG NOD mice Lobe 1 + Lobe 2 parasite mean percentage (± s.d.)**	**Lobe 1 + Lobe 2 mean parasite number (± s.d.)**
NF54mCherry + AV27 M4, Lobe 1	1A	18	10	141	14	2	16	**9**	**93/7**	**AV27: 94% NF54: 6% (± 1.4%)**	**AV27: 94.8 (± 21.2) NF54: 6.9 (± 1.4)**
	2A	17	10	133	12	2	14				
	3B	14	10	110	6	0	6				
	4B	15	11	130	9	0	9				
	5B	12	9	85	8	0	8				
	1E	18	15	212	12	1	13				
	2E	16	10	126	12	0	12				
	3F	15	11	130	9	0	9				
	4F	18	10	141	19	1	20				
	5F	14	12	132	10	2	12				
	**TOT**			**1339**	**111**	**8**	**119**				
NF54mCherry + AV27 M4, Lobe 2	1O	9	15	106	11	1	12	**8**	**93/7**		
	2O	9	17	120	9	0	9				
	3O	10	15	118	12	1	13				
	1P	9	14	99	8	0	8				
	2P	11	15	130	6	0	6				
	3P	11	16	138	12	2	14				
	1Q	12	17	160	10	1	11				
	2Q	14	18	198	13	1	14				
	**TOT**			**1068**	**81**	**6**	**87**				
NF54mCherry + AV27 M5, Lobe 1	1L	16	10	126	39	2	41	**27**	**94/6**		**AV27: 262 (± 45.2) NF54: 14.4 (± 5.6)**
	2L	17	10	133	54	5	59				
	3L	17	10	133	58	2	60				
	4M	15	10	118	35	3	38				
	5M	15	10	118	38	4	42				
	6N	15	10	118	36	1	37				
	7N	26	20	408	36	2	38				
	**TOT**			**1154**	**296**	**19**	**315**				
NF54mCherry + AV27 M5, Lobe 2	1U	9	12	85	28	2	30	**26**	**95.5/4.5**		
	2U	9	11	78	22	1	23				
	3U	12	15	141	22	2	24				
	1V	11	16	138	27	0	27				
	2V	10	17	133	28	1	29				
	3V	8	17	107	33	3	36				
	1Z	8	12	75	21	1	22				
	2Z	10	10	79	23	0	23				
	3Z	9	16	113	28	1	29				
	**TOT**			**949**	**232**	**11**	**243**				
NF54mCherry + AV66 M6, Lobe 1	1C	19	13	194	22	3	25	**14**	**90/10**	**AV66: 88% NF54: 12% (± 3.9%)**	**AV66: 84 (± 128) NF54: 8.7 (± 15.5)**
	2C	15	10	118	13	1	14				
	3D	20	12	188	25	4	29				
	4D	20	13	204	27	4	31				
	1G	21	13	214	30	4	34				
	2G	22	14	242	29	4	33				
	3H	20	12	188	30	2	32				
	4H	18	13	184	21	1	22				
	5H	17	15	200	17	2	19				
	**TOT**			**1732**	**214**	**25**	**239**				
NF54mCherry + AV66 M6, Lobe 2	NL16.1	11	5	43	1	0	1	**9**	**92/8**		
	NL16.2	9	6	42	1	1	2				
	NL16.3	10	4	31	6	0	6				
	NL16.4	10	7	55	5	0	5				
	NL17.1	11	6	52	3	0	3				
	NL17.2	10	5	39	3	0	3				
	NL17.3	9	7	49	5	0	5				
	NL17.4	9	5	35	2	0	2				
	NL17.5	9	5	35	7	2	9				
	**TOT**			**383**	**33**	**3**	**36**				
NF54mCherry + AV66 M7, Lobe 1	NL11.1	10	12	94	20	3	23	**15**	**87/13**		**AV66: 109.4 (± 7.8) NF54: 18.8 (± 4.2)**
	NL11.2	11	19	164	13	0	13				
	NL11.3	7	13	71	15	1	16				
	NL11.4	10	14	110	7	3	10				
	NL12.1	10	16	126	18	5	23				
	NL12.2	12	10	94	10	3	13				
	NL12.3	10	19	149	21	1	22				
	**TOT**			**809**	**104**	**16**	**120**				
NF54mCherry + AV66 M7, Lobe 2	NL13.1	11	11	95	15	5	20	**21**	**84/16**		
	NL13.2	11	10	86	15	2	17				
	NL14.1	12	12	113	17	6	23				
	NL14.2	11	11	95	15	1	16				
	NL14.3	10	10	79	20	2	22				
	NL15.1	12	12	113	16	3	19				
	NL15.2	11	9	78	17	3	20				
	**TOT**			**659**	**115**	**22**	**137**				

Supplemental tables 12, 13, 14 and 15. Class I immunopeptidome for Pf3D7 (proxy for NF54) and MAL31. For each parent, the total number of strong-binding epitopes present in each chromosome was determined separately for epitopes of 8 amino acids in length (8-mers), 9-mers, 10-mers and 11-mers. A large number of epitopes are present multiple times in a chromosome (duplicates). For each unique epitope (equaling the total number minus duplicates), the number present in both parental strains is shown in the last column.

Supplemental Table 12. Parental 8-mers

	Class I immunopeptidome (epitopes of 8-amino-acid)		
**Chromosome**	**Pf3D7/NF54**<br>**Total (duplicates)**	**MAL31**<br>**Total (duplicates)**	**Unique epitope sequences shared by both parental strains**
Chrom 1	2,792 (582)	2,535 (572)	1,518
Chrom 2	5,108 (1,079)	4,317 (880)	2,729
Chrom 3	6,444 (1,378)	5736 (1,287)	3,576
Chrom 4	6,351 (1,464)	5,834 (1,464)	3,276
Chrom 5	8,018 (1,626)	7,275 (1,525)	4,530
Chrom 6	8,124 (1,667)	7,181 (1,479)	4,507
Chrom 7	8,315 (1,670)	7,337 (1,570)	4,446
Chrom 8	8,045 (1,618)	7,363 (1,598)	4,464
Chrom 9	8754 (1,781)	7,754 (1,598)	4,876
Chrom 10	9,472 (2,130)	8,390 (1,930)	5,119
Chrom 11	11,703 (2,411)	10,505 (2,188)	6,586
Chrom 12	13,416 (2,807)	12,158 (2,646)	7,405
Chrom 13	18,215 (3,747)	16,327 (3,525)	10,135
Chrom 14	21,183 (4,307)	18,790 (3,889)	11,870
**TOTAL**	**135,940 (28,267)**	**121,502 (26,151)**	**75,037**

Supplemental Table 13. Parental 9-mers

Chromosome	Class I immunopeptidome (epitopes of 9-amino-acid)		
	Pf3D7/NF54 Total (duplicates)	MAL31 Total (duplicates)	Unique epitope sequences shared by both parental strains
Chrom 1	28,543 (15,429)	24,799 (12,755)	10,503
Chrom 2	53,913 (29,977)	45,477 (23,585)	19,604
Chrom 3	65,144 (35,747)	56,622 (29,272)	24,512
Chrom 4	66,740 (36,804)	59,426 (31,078)	23,628
Chrom 5	85,488 (46,914)	75,024 (38,750)	32,435
Chrom 6	84,043 (46,047)	74,033 (38,309)	31,649
Chrom 7	87,374 (47,584)	76,333 (39,187)	32,157
Chrom 8	81,972 (44,555)	72,263 (37,196)	30,881
Chrom 9	91,119 (49,801)	78,354 (40,160)	34,479
Chrom 10	99,656 (56,175)	86,354 (46,043)	36,138
Chrom 11	119,434 (65,257)	104,374 (53,358)	45,726
Chrom 12	139,476 (76,270)	123,303 (63,195)	52,614
Chrom 13	188,337 (102,701)	163,680 (84,120)	71,536
Chrom 14	220,052 (120,584)	188,099 (96,309)	82,728
**TOTAL**	**1,411,291 (773,845)**	**1,228,141 (633,317)**	**528,590**

Supplemental Table 14. Parental 10-mers

Chromosome	Class I immunopeptidome (epitopes of 10-amino-acid)		
	**Pf3D7/NF54 Total (duplicates)**	**MAL31 Total (duplicates)**	**Unique epitope sequences shared by both parental strains**
Chrom 1	11,082 (582)	10,551 (572)	5,143
Chrom 2	20,109 (1,079)	18,483 (880)	9,352
Chrom 3	24,643 (1,378)	23,282 (1,287)	11,897
Chrom 4	25,850 (1,464)	25,144 (1,464)	11,406
Chrom 5	32,321 (1,626)	31,098 (1,525)	15,830
Chrom 6	31,661 (1,667)	30,292 (1,479)	15,176
Chrom 7	33,253 (1,670)	31,738 (1,570)	15,546
Chrom 8	30,961 (1,618)	29,661 (1,598)	14,882
Chrom 9	33,832 (1,781)	32,023 (1,598)	16,422
Chrom 10	37,551 (2,130)	35,473 (1,930)	17,663
Chrom 11	45,987 (2,411)	43,836 (2,188)	22,326
Chrom 12	53,598 (2,807)	51,712 (2,646)	25,625
Chrom 13	71,932 (3,747)	68,386 (3,525)	34,773
Chrom 14	83,622 (4,307)	77,875 (3,889)	40,233
**TOTAL**	**536,402 (28,267)**	**509,554 (26,151)**	**256,274**

Supplemental Table 15. Parental 11-mers

Chromosome	Class I immunopeptidome (epitopes of 11-amino-acid)		
	Pf3D7/NF54 Total (duplicates)	MAL31 Total (duplicates)	Unique epitope sequences shared by both parental strains
Chrom 1	2,364 (582)	6,048 (572)	1,525
Chrom 2	4,271 (1,079)	10,340 (880)	2,770
Chrom 3	5,352 (1,378)	13,094 (1,287)	3,564
Chrom 4	5,589 (1,464)	13,956 (1,464)	3,384
Chrom 5	6,765 (1,626)	17,064 (1,525)	4,595
Chrom 6	6,707 (1,667)	17,500 (1,479)	4,496
Chrom 7	7,209 (1,670)	17,962 (1,570)	4,616
Chrom 8	6,69 (1,618)	16,739 (1,598)	4,428
Chrom 9	7,290 (1,781)	17,848 (1,598)	4,910
Chrom 10	7,866 (2,130)	20,520 (1,930)	5,187
Chrom 11	9,860 (2,411)	24,577 (2,188)	6,697
Chrom 12	11,173 (2,807)	28,828 (2,646)	7,413
Chrom 13	15,489 (3,747)	38,095 (3,525)	10,461
Chrom 14	17,764 (4,307)	43,614 (3,889)	11,924
**TOTAL**	**114,390 (28,267)**	**286,185 (26.151)**	**75,970**

Supplemental Tables 16, 17 and 18. Class I immunopeptidome for the hybrid AV27. For each chromosome, the parental source of each segment was determined. For each segment, the total number of epitopes present in respective parent was determined. For each unique epitope sequence, we determined the number present also in the same chromosome of the second parent.

Supplemental Table 16. AV27 8-mers

Chromosome	Class I immunopeptidome (epitopes of 8-amino-acid)			
	3D7/NF54 Segments Total (duplicates)	Unique 3D7/NF54 epitope sequences also present in MAL31	MAL31 Segments Total (duplicates)	Unique epitope sequences also present in Pf3D7/NF54
**AV27_01**	n/a	n/a	2,535 (572)	1,518
**AV27_02**	2,623 (565)	1,412	1,829 (377)	1,169
**AV27_03**	1,852 (436)	946	4,088 (878)	2,591
**AV27_04**	5,122 (1,112)	2,742	1,065 (254)	537
**AV27_05**	4,079 (838)	2,296	3,588 (756)	2,202
**AV27_06**	1,930 (355)	1,091	5,466 (1,107)	3,433
**AV27_07**	n/a	n/a	7,337 (1,579)	4,446
**AV27_08**	n/a	n/a	7,363 (1,598)	4,464
**AV27_09**	8,754 (1,781)	4,876	n/a	n/a
**AV27_10**	7,110 (1,512)	3,942	2,088 (596)	1,149
**AV27_11**	3,798 (776)	2,142	6,904 (1,419)	4,388
**AV27_12**	6,427 (1,322)	3,571	6,388 (1,445)	3,810
**AV27_13**	12,531 (2,531)	7,007	5,114 (1,149)	3,103
**AV27_14**	19,209 (3,878)	10,914	1,487 (297)	956
**TOTAL**	**73,435 (15,106)**	**40,939**	**55,252 (12,018)**	**33,766**

Supplemental Table 17. AV27 10-mers

Chromosome	Class I immunopeptidome (epitopes of 10-amino-acid)			
	3D7/NF54 Segments Total (duplicates)	Unique epitope sequences also present in MAL31	MAL31 Segments Total (duplicates)	Unique epitope sequences also present in Pf3D7/NF54
**AV27_01**	n/a	n/a	10,551 (4,166)	5,143
**AV27_02**	10,242 (4,169)	4,840	8,076 (3,217)	4,021
**AV27_03**	6,879 (2,808)	3,139	16,679 (6,322)	8,671
**AV27_04**	20,749 (8,504)	9,472	4,693 (1,772)	1,949
**AV27_05**	16,207 (6,503)	7,929	15,336 (5,953)	7,810
**AV27_06**	7,584 (2,986)	3,621	23,012 (8,870)	11,611
**AV27_07**	n/a	n/a	31,738 (12,230)	15,546
**AV27_08**	n/a	n/a	29,661 (11,354)	14,882
**AV27_09**	33,832 (13,561)	16,422	n/a	n/a
**AV27_10**	27,931 (11,368)	13,595	8,736 (3,812)	3,953
**AV27_11**	15,001 (6,093)	7,346	28,763 (10,968)	14,752
**AV27_12**	25,353 (10,070)	12,430	27,242 (10,698)	13,094
**AV27_13**	49,409 (19,717)	23,916	21,507 (8,476)	10,780
**AV27_14**	75,872 (30,344)	36,961	6,294 (2,402)	3,272
**TOTAL**	**289,059 (116,123)**	**139,671**	**232,288 (90,240)**	**115,484**

Supplemental Table 18. AV27 11-mers

Chromosome	Class I immunopeptidome (epitopes of 11-amino-acid)			
	3D7/NF54 Segments Total (duplicates)	Unique epitope sequences also present in MAL31	MAL31 Segments Total (duplicates)	Unique epitope sequences also present in Pf3D7/NF54
**AV27_01**	n/a	n/a	6,048 (2,150)	1,525
**AV27_02**	2,097 (593)	1,400	4,561 (1,552)	1,223
**AV27_03**	1,590 (491)	999	9,327 (3,194)	2,538
**AV27_04**	4,490 (1,371)	2,784	2,678 (901)	606
**AV27_05**	3,368 (959)	2,304	8,531 (3,021)	2,266
**AV27_06**	1,579 (420)	1,088	13,284 (4,649)	3,427
**AV27_07**	n/a	n/a	17,962 (6,137)	4,616
**AV27_08**	n/a	n/a	16,739 (5,685)	4,428
**AV27_09**	7,290 (2,097)	4,910	n/a	n/a
**AV27_10**	5,969 (1,743)	4,038	5,605 (2,504)	1,121
**AV27_11**	3,188 (910)	2,185	16,125 (5,474)	4,424
**AV27_12**	5,379 (1,543)	3,624	15,126 (5,205)	3,765
**AV27_13**	10,611 (3,019)	7,155	11,965 (4,156)	3,290
**AV27_14**	16,134 (4,626)	10,971	3,516 (1,206)	953
**TOTAL**	**61,695 (17,772)**	**41,458**	**131,467 (45,834)**	**34,182**

Supplemental Figure 1. Expression analysis and characterization of the NF54^{mCherry} transgenic line. Live fluorescence imaging of **(A)** asexual (rings, trophozoites and schizonts) and **(B)** gametocyte (stages II–V) stages of the NF54^{mCherry} clone 2B5, showing robust expression of the mCherry reporter. Nuclear DNA is visualized with Hoechst (blue). Scale bars, 5 µm. **(C)** NF54^{mCherry} displays infectivity in *Anopheles stephensi* mosquitoes comparable to the parental NF54 strain. Data represent median salivary gland sporozoite loads from 25 mosquitoes per biological replicate (n = 3). Statistical comparisons were performed using two-way ANOVA.

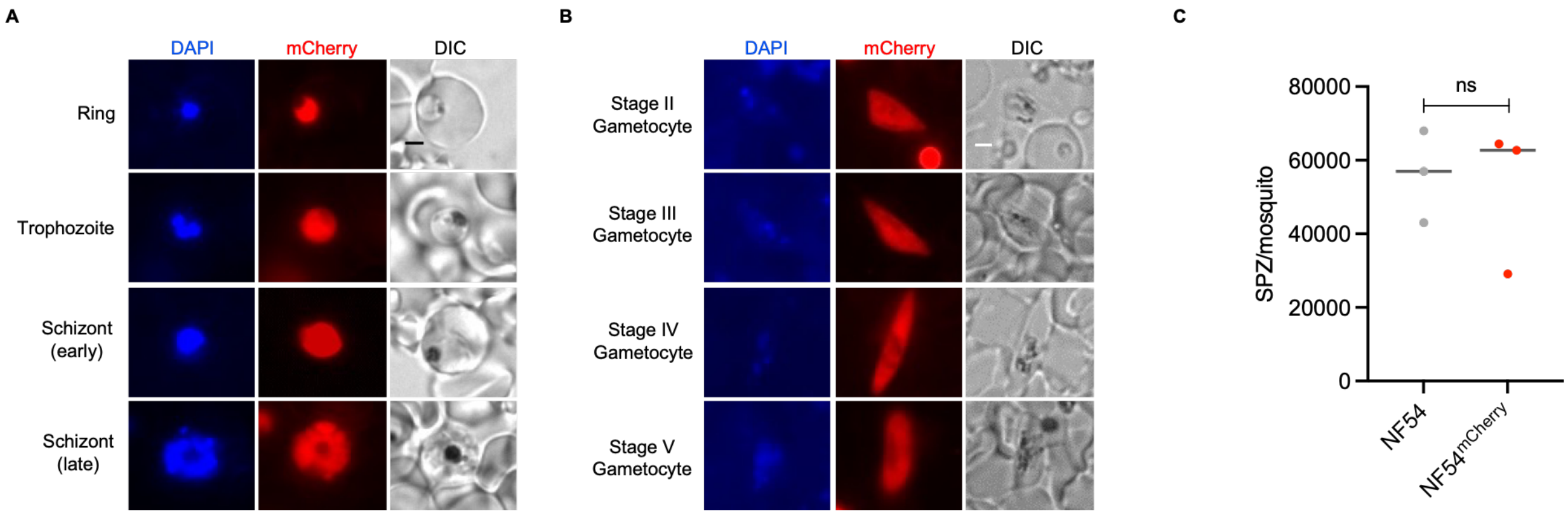

Supplemental Figure 2. *P. falciparum* hybrid clone AV23 shows high frequency of oocyst melanization at day 7 post infectious feed. (A) Midgut of mosquito without melanized oocysts. **(B)** *P. falciparum* (NF54 strain), with few melanized oocysts. **(C)** *P. falciparum* AV23 (NF54 x MAL31) hybrid line. Black dots are melanized oocysts (arrow) in *A. stephensi* fresh unstained midgut at 16 dpi. Magnification:100x.

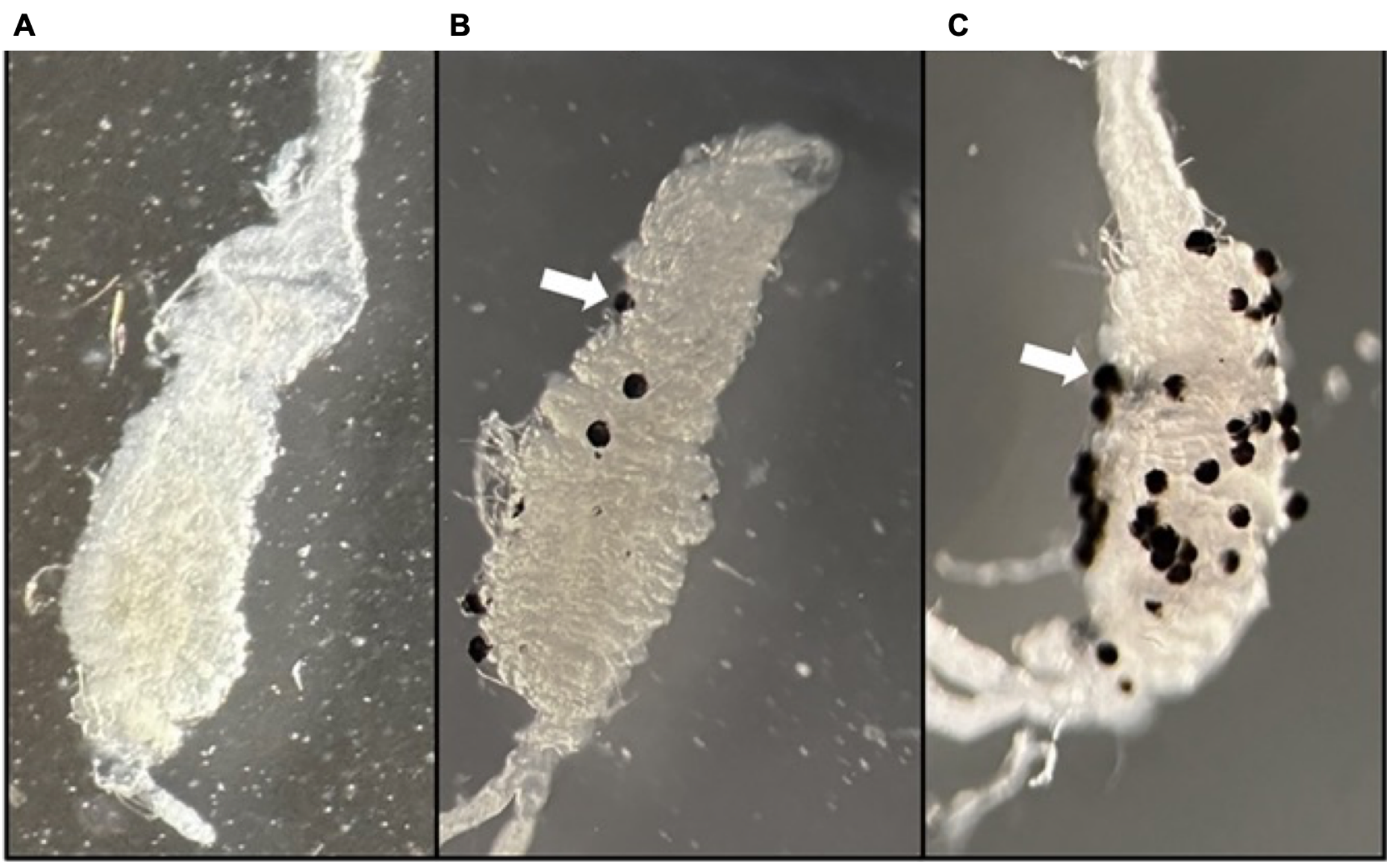

Supplemental Figure 3. Comparison of *P. falciparum* sporozoite (PfSPZ) loads across parasite strains over a 2-year period. Box and whisker plots display the distribution of PfSPZ per mosquito for MAL31 (n = 17), NF54 (n = 12), and AV27 (n = 11). Boxes represent the interquartile range (IQR), and whiskers represent the minimum and maximum ranges. Individual data points for each mosquito are overlaid as circles. Horizontal lines within the boxes indicate the median values, with exact numerical medians highlighted adjacent to each plot (MAL31: 3,134; NF54: 62,388; AV27: 78,179). White diamonds represent the mean values for each strain. Statistical significance was determined using a Mann-Whitney U test, with corresponding *P*-values displayed above the brackets ($p < 0.0001$ for MAL31 vs. NF54 and MAL31 vs. AV27; $p = 0.5658$ for NF54 vs. AV27).

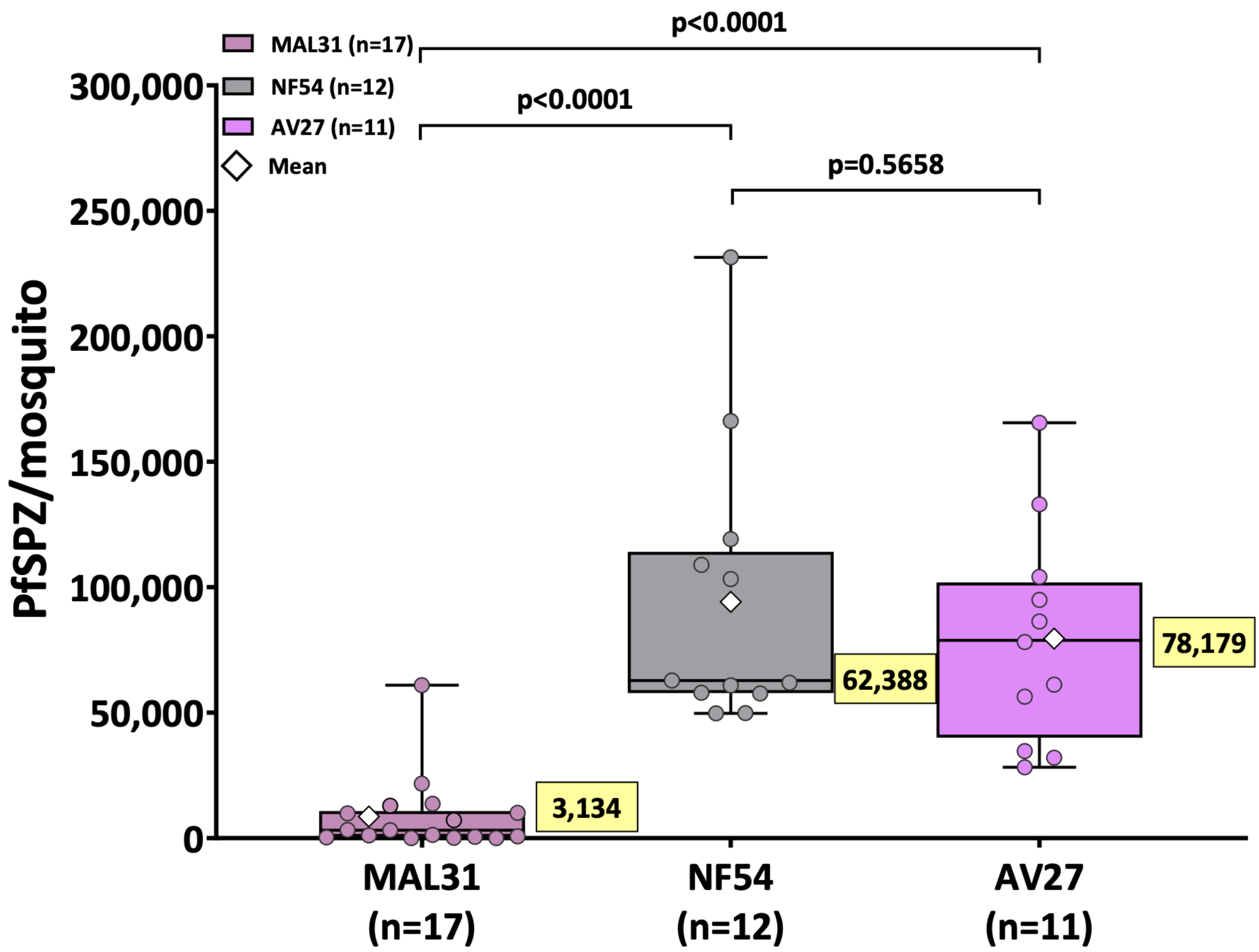

Supplement: Supplementary file 1 [file jciinsight-11-207272-s177.pdf]
